# Effects of Sorafenib on Intra-Tumoral Interstitial Fluid Pressure and Circulating Biomarkers in Patients with Refractory Sarcomas (NCI Protocol 6948)

**DOI:** 10.1371/journal.pone.0026331

**Published:** 2012-02-07

**Authors:** Chandrajit P. Raut, Yves Boucher, Dan G. Duda, Jeffrey A. Morgan, Richard Quek, Marek Ancukiewicz, Johanna Lahdenranta, J. Paul Eder, George D. Demetri, Rakesh K. Jain

**Affiliations:** 1 Department of Surgery, Brigham and Women's Hospital and Dana-Farber Cancer Institute, Harvard Medical School, Boston, Massachusetts, United States of America; 2 Department of Radiation Oncology, Massachusetts General Hospital, Boston, Massachusetts, United States of America; 3 Department of Medical Oncology, Brigham and Women's Hospital and Dana-Farber Cancer Institute, Harvard Medical School, Boston, Massachusetts, United States of America; 4 Harvard Medical School, Boston, Massachusetts, United States of America; Ohio State University, United States of America

## Abstract

**Purpose:**

Sorafenib is a multi-targeted tyrosine kinase inhibitor with therapeutic efficacy in several malignancies. Sorafenib may exert its anti-neoplastic effect in part by altering vascular permeability and reducing intra-tumoral interstitial hypertension. As correlative science with a phase II study in patients with advanced soft-tissue sarcomas (STS), we evaluated the impact of this agent on intra-tumor interstitial fluid pressure (IFP), serum circulating biomarkers, and vascular density.

**Patients and Methods:**

Patients with advanced STS with measurable disease and at least one superficial lesion amenable to biopsy received sorafenib 400 mg twice daily. Intratumoral IFP and plasma and circulating cell biomarkers were measured before and after 1–2 months of sorafenib administration. Results were analyzed in the context of the primary clinical endpoint of time-to-progression (TTP).

**Results:**

In 15 patients accrued, the median TTP was 45 days (range 14–228). Intra-tumoral IFP measurements obtained in 6 patients at baseline showed a direct correlation with tumor size. Two patients with stable disease at two months had post-sorafenib IFP evaluations and demonstrated a decline in IFP and vascular density. Sorafenib significantly increased plasma VEGF, PlGF, and SDF1α and decreased sVEGFR-2 levels. Increased plasma SDF1α and decreased sVEGFR-2 levels on day 28 correlated with disease progression.

**Conclusions:**

Pretreatment intra-tumoral IFP correlated with tumor size and decreased in two evaluable patients with SD on sorafenib. Sorafenib also induced changes in circulating biomarkers consistent with expected VEGF pathway blockade, despite the lack of more striking clinical activity in this small series.

**Trial Registration:**

ClinicalTrials.gov NCT00330421

## Introduction

The bi-aryl urea sorafenib was initially developed as an inhibitor of *c-raf* and mutant (V599E) *b-raf in vitro* [1]. The *ras/raf* signaling pathway is an important mediator of responses to growth signals and angiogenic factors. However, sorafenib also inhibits several receptor tyrosine kinases that may be involved in tumor angiogenesis and progression, e.g., human and murine vascular endothelial growth factor receptor-2 (VEGFR-2), VEGFR-3, platelet-derived growth factor receptor-beta (PDGFR-β), Flt3, and c-KIT [2], [3], [4]. Indeed, in human tumor xenografts, sorafenib induced a dramatic reduction in tumor neo-vascularization. These data suggest that sorafenib may have antineoplastic activity through multiple mechanisms, directly by targeting cell proliferation/survival dependent on activation of the MAPK pathway and by inhibiting tumor angiogenesis through inhibition of VEGFR-2, VEGFR-3, and/or PDGFR-β. Sorafenib has been approved by the United States Food and Drug Administration for the treatment of patients with renal cell carcinoma and hepatocellular carcinoma, and it remains under investigation in several other solid tumors and hematologic malignancies.

Studies from our group and others have shown that the intra-tumoral interstitial fluid pressure (IFP) in human sarcomas, melanomas, and carcinomas (including colon, breast, lung, head and neck, cervix) is significantly higher than in normal tissues [5], [6], [7], [8], [9], [10], [11], [12], [13], [14], [15], [16], [17]. Increased permeability of blood vessels, impaired interstitial and lymphatic drainage, and compression of blood vessels by tumor cells growing in a confined space are major causes of intra-tumoral interstitial hypertension [18]. VEGF and PDGF signaling pathways have previously been etiologically related to tumor interstitial hypertension. Antibody blockade of VEGFR-2 reduces both tumor vascular permeability and IFP and increases both the transvascular pressure gradient and penetration of small tracers into solid tumors [19], [20]. Similarly, the inhibition of PDGF signaling (by DNA aptamers, imatinib, etc.) may reduce tumor IFP, increase tumor uptake of chemotherapy agents, and enhance their therapeutic effects [21], [22], [23]. However, responses to antiangiogenic agents are invariably transient, and the escape mechanisms remain elusive [24].

Using study drug supplied by the NCI Cancer Therapy Evaluation Program (CTEP), we conducted a phase II trial of sorafenib in patients with advanced soft tissue sarcomas (STS), with the aim of exploring whether sorafenib administration is associated with mechanistically-related changes in intra-tumoral IFP and vascular density as well as circulating biomarkers of angiogenesis.

## Methods

### Trial Design

**Figure 1:**
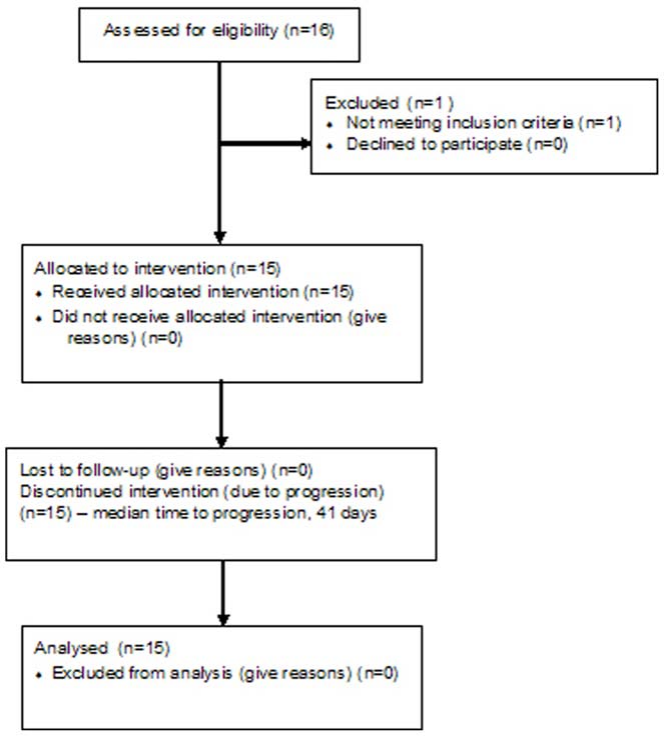
Flow Diagram.

The protocol for this trial and supporting CONSORT checklist are available as supporting information; see Checklist S1 and Protocol S1. This phase II study (**Figure 1**) was approved by the Institutional Review Board of the Dana-Farber/Harvard Cancer Center for patients with metastatic or inoperable soft tissue sarcomas with no available curative or definitive survival-prolonging palliative therapy. Additional eligibility criteria included: at least one site of measurable disease by radiologic imaging, at least one superficial palpable tumor (>1 cm) with no overlying viscera amenable to biopsy, age≥18 years, Eastern Cooperative Oncology Group (ECOG) performance status ≤2, and no prior sorafenib therapy. Written informed consent was obtained from all study participants. Sorafenib was administered at 400 mg twice daily continuously in cycles arbitrarily denoted as 28 days in length.

### Data Acquisition

Evaluations included physical examination, laboratory data, documentation of ECOG performance status, CT or MRI imaging (at the discretion of the treating physicians), and electrocardiogram. Each of these evaluations was performed prior to initial sorafenib administration, every one to four weeks (depending on cycle) while on study, and one month after the last dose of sorafenib was administered. Imaging was performed every other month while on study. Adverse events and toxicities were assessed on schedule every one to four weeks (depending on cycle) and one month after the last dose of sorafenib was administered. Pharmacokinetic data were measured on days 28 and 56.

### Evaluation of Biomarkers

***Histology.*** Biopsies were available from 3 patients at baseline and after 28 or 56 days of sorafenib therapy. Five µm-thick sections were cut from the formalin-fixed, paraffin embedded blocks and a double immunostaining procedure was performed with CD31 (Dako N1596, Carpentria, CA) and α-smooth muscle actin (α-SMA; Dako M0850) antibodies. In brief, the CD31 antibody was incubated at room temperature for 1 hour. Slides were then washed and incubated in secondary antibody (DAKO EnVision anti-mouse, K4007) for 30 min and developed with DAB. Slides were then blocked with EnVision doublestain block for 5 min and incubated overnight with the α-SMA antibody. After washes, slides were incubated in secondary antibody (DAKO Doublestain AP Polymer) for 30 min, washed, and developed with Fast Red. Slides were counterstained with hematoxylin and coverslipped with Faramount. To determine the percentage of proliferating cancer cells, immunostaining was also performed with a Ki67 antibody (Dako N1633).

***Circulating Biomarkers.*** Peripheral blood was collected in EDTA-containing vacutainers from patients enrolled in this study at baseline (prior to sorafenib administration) and 28 days following the first dose of sorafenib. Blood was available from 14 patients at baseline and 10 patients at 28 days. Plasma analysis was carried out for circulating VEGF, placental growth factor (PlGF), soluble VEGFR-1 (sVEGFR-1), basic fibroblast growth factor (bFGF), interleukin-1β (IL-1β), IL-6, IL-8, and tumor necrosis factor-alpha (TNF-α) using multiplex ELISA plates from Meso-Scale Discovery, as well as for sVEGFR-2 and stromal cell-derived factor-1-alpha (SDF1α) using kits from R&D Systems [25]. Every sample was run in duplicate. Blood-circulating CD34^+^CD45^dim^ progenitor cells (CPCs) and VEGFR-2^+^CD45^+^ monocytes were enumerated in fresh samples using a standard flow cytometry protocol [26]. The quantitative analysis endpoint was the change in the fraction of CPCs or VEGFR-2^+^ monocytes within the mononuclear blood cell population after sorafenib treatment. Percent values were obtained pre-treatment and at day 28 after the first dose of sorafenib.

***Intra-Tumoral IFP.*** Intra-tumoral IFP was measured intraoperatively as previously described [9] prior to administration of the first dose of sorafenib and, in the absence of progression or drug intolerance, repeated on study day 28 or 56. In brief, to measure IFP, a 23-gauge needle with a 2 mm side hole at 5 mm from the tip was used. Nylon filaments (6-0 Ethilon) were placed in the needle. To take the pressure measurements, the needle and tubing filled with sterile heparinized saline were connected to a disposable pressure transducer and an electronic data acquisition and recording system (AdInstruments Inc, Colorado Springs, CO). The needle and tubing were gas sterilized before use. The calibration of the pressure transducer was verified by applying pressures of 10, 20, and 40 mm Hg before each IFP measurement. With the patient in supine position, the needle was inserted into the tumor center and the IFP was recorded. Stable pressure measurements with a good fluid communication between the tumor interstitial space and needle were considered valid. The IFP was measured in 2 to 3 different locations within the tumor. All IFP measurements were performed in superficial tumors under local anesthetic.

### Data and Statistical Analyses

The correlative scientific endpoints of this trial included measurements of changes in circulating biomarkers and IFP, radiographic responses, toxicity, and pharmacokinetics. The primary clinical endpoint was time-to-progression (TTP), measured from date of registration to date of radiographic progression. Response and progression were evaluated using the Response Evaluation Criteria in Solid Tumors (RECIST) [27]. Radiographic response was defined as percentage change in tumor size.

Biomarker changes from baseline were tested using the exact paired Wilcoxon test [28]. Missing measurements were excluded from analysis. In exploratory studies, we tested the correlation of baseline biomarker or biomarker changes at day 28 with pre-treatment tumor size, best tumor response (SD), or radiographic tumor response (as ordinal variables) using Kendall's τβ coefficients [29].

## Results

### Demographic Data and Clinical Effects of Sorafenib

Patient and tumor characteristics are listed in **Table 1**. Fourteen of 15 patients (93.3%) had received prior chemotherapy and/or radiation therapy. No patients experienced a complete or partial radiographic response by RECIST. Stable disease (SD) was observed in 8 patients (53%) for a median 72 days (range 45–228 days). Progressive disease was observed as the “best response” in the remaining 7 patients (47%). Median TTP for the entire cohort was 45 days (range 14 to 228 days). Clinical outcomes did not appear to correlate with any specific histology; the 4 patients with TTP>80 days had 4 different sarcoma histologies (desmoplastic small round cell tumor, leiomyosarcoma, myxofibrosarcoma, and synovial sarcoma).

**Table 1:** Clinical characteristics.

Patient characteristics	Number (%)
Median age	59 years (range, 30–84 years)
Sex	
Male	9 (60)
Female	6 (40)
ECOG* Performance Status	
0	8 (53.3)
1	7 (46.7)
Histology	
Angiosarcoma	1 (6.7)
Desmoplastic small round cell tumor	1 (6.7)
Gastrointestinal stromal tumor	1 (6.7)
Leiomyosarcoma	4 (26.7)
Liposarcoma	1 (6.7)
Malignant diffuse-type giant cell tumor	1 (6.7)
Malignant peripheral nerve sheath tumor	1 (6.7)
Malignant phyllodes tumor	1 (6.7)
Myxofibrosarcoma	2 (13.3)
Synovial sarcoma	2 (13.3)
Primary site	
Upper extremity	1 (6.7)
Lower extremity	1 (6.7)
Trunk	12 (80.0)
Pelvis	1 (6.7)

*ECOG, Eastern Cooperative Oncology Group.

### Safety

Adverse events probably or definitely related to treatment are listed in **Table 2**. No Grade 4 toxicities were noted. The most commonly observed adverse events were hand-foot syndrome (7 patients), fatigue (3), mucositis/stomatitis (4), and hypertension (3) (**Table 2**). Of note, sorafenib administration transiently increased the number of red blood cells and blood hemoglobin at day 14 (**Table S1**).

**Table 2:** Adverse events after sorafenib treatment in advanced soft tissue sarcoma patients: number of episodes/number of affected patients (percentage).

Toxicity	Grade 1 (%)	Grade 2 (%)	Grade 3 (%)	Grade 4 (%)	Any Grade (%)
Hand-foot syndrome	13/6 (40.0)	5/2 (13.3)	3/3 (20.0)	0	21/7 (46.7)
Rash/desquamation	4/1 (6.7)	2/1 (6.7)	3/1 (6.7)	0	8/1 (6.7)
Fatigue	5/3 (20.0)	0	0	0	5/3 (20.0)
Mucositis/stomatitis	5/4 (26.7)	0	0	0	5/4 (26.7)
Hypertension	2/2 (13.3)	0	2/1 (6.7)	0	4/3 (20.0)
Extremity pain	4/1 (6.7)	0	0	0	4/1 (6.7)
Erythema multiforme	2//1 (6.7)	1/1 (6.7)	0	0	3/2 (13.3)
Skin – other	2/2 (13.3)	0	1/1 (6.7)	0	3/2 (13.3)
Hemoglobin	0	2/1 (6.7)	0	0	2/1 (6.7)
Anorexia	2/1 (6.7)	0	0	0	2/1 (6.7)
Bilirubin	2/1 (6.7)	0	0	0	2/1 (6.7)
Oral cavity – pain	2/1 (6.7)	0	0	0	2/1 (6.7)
Platelets	1/1 (6.7)	0	0	0	1/1 (6.7)
Fever without neutropenia	1/1 (6.7)	0	0	0	1/1 (6.7)
Alopecia	1/1 (6.7)	0	0	0	1/1 (6.7)
Pruritis	0	1/1 (6.7)	0	0	1/1 (6.7)
Dehydration	0	0	1/1 (6.7)	0	1/1 (6.7)
Diarrhea	0	1/1 (6.7)	0	0	1/1 (6.7)
Alkaline phosphatase	1/1 (6.7)	0	0	0	1/1 (6.7)
Muscle - pain	1/1 (6.7)	0	0	0	1/1 (6.7)

### Analyses of Circulating Biomarkers

As a mechanistic pharmacodynamic assessment of sorafenib administration, we measured circulating levels of angiogenic biomarkers before and after sorafenib dosing, compared baseline biomarker levels with baseline tumors characteristics, and correlated baseline biomarker levels or changes in biomarker levels with radiographic responses. Sorafenib treatment induced significant increases in plasma circulating VEGF, PlGF, IL-8, and SDF1α and decreases in sVEGFR2, but not other angiogenic and inflammatory biomarkers (bFGF, sVEGFR-1, TNF-α, IL-6, CPCs or VEGFR-2^+^ monocytes) (**Table 3** and not shown). IL-1β concentration was undetectable in the majority of plasma samples. Higher baseline plasma concentration of IL-6 correlated with larger baseline tumor size (p<0.05, **Table 4**). Lower baseline plasma PlGF levels correlated with improved radiographic response after sorafenib dosing (p<0.05, **Table 4**). With respect to biomarkers that changed after one cycle of sorafenib (day 28), the decrease in plasma sVEGFR-2 correlated with both SD and trend toward improved radiographic response, and the increase in plasma SDF1α correlated with worse radiographic tumor response (p<0.05; **Table 4**). In the samples from patients with SD who were on-study and evaluable at 56 days, there were no statistically significant differences in the measured biomarkers, likely due to the small sample size (n = 4; data not shown).

**Table 3:** Plasma biomarker concentration (pg/ml) before (pre-treament) and after 28 days after sorafenib treatment.

	Pre-Treatment	Day 28	
Plasma Biomarker	(N = 14)	(N = 10)	*P*-value
**VEGF**	140 [87,161]	214 [154,311]	0.002
**bFGF (pg/ml)**	36 [19,68]	29 [15,86]	0.19
**PlGF**	22 [17,34]	52 [40,62]	0.002
**sVEGFR-1 (pg/ml)**	112 [99,142]	83 [66,93]	0.38
**sVEGFR-2**	6212 [5826–7207]	4781 [3942–5484]	0.002
**SDF1α**	2306 [2218,2582]	2705 [2531,3472]	0.0039
**IL-6 (pg/ml)**	5.8 [3.9,17.2]	12 [5,33]	0.13
**IL-8**	5.7 [4.3,14.5]	7.1 [5.6,22.2]	0.0059
**TNF-α (pg/ml)**	9.2 [7.4,11.8]	9.2 [7.4,14.8]	0.11
**CPCs (% of PBMCs)**	0.050 [0.030,0.074]	0.057 [0.029,0.075]	0.20

Data are shown as medians and interquartile ranges (in square brackets) compared to baseline levels. *P*-values are from Wilcoxon test.

VEGF, vascular endothelial growth factor; bFGF, basic fibroblast growth factor; PlGF, placental growth factor; sVEGFR-1, soluble VEGF receptor-1; sVEGFR-2, soluble VEGF receptor-2; SDF1α, stromal cell-derived factor-1-alpha; IL-6, interleukin-6; IL-8, interleukin-8; TNF-α, tumor necrosis factor-alpha, CPCs, circulating progenitor cells; PBMC, peripheral blood mononuclear cells.

**Table 4:** Analysis of correlation between baseline biomarker and biomarker change at day 28 with (i) pre-treatment tumor size, (ii) best tumor response, and (iii) radiographic tumor response after sorafenib treatment in advanced STS patients (Kendall's *τ*β with 95% CI).

Kendall's *τ*β	Pre-Treatment Size	Response (SD)	Radiographic Response
**Baseline IFP (N = 6)** 1	0.87 [0.56,1.17]	−0.43 [−0.91,0.05]	−0.20 [−0.63,0.23]
*P*-value	0.017	0.40	0.82
**Baseline IL-6 (N = 14)** 1	0.42 [0.10,0.74]	0.11 [−0.23,0.45]	0.09 [−0.36,0.54]
*P*-value	0.037	0.70	0.74
**Baseline PlGF (N = 14)** 1	0.31 [−0.03,0.65]	−0.39 [−0.64,−0.14]	−0.61 [−0.94,−0.27]
*P*-value	0.12	0.11	0.0054
**Change in sVEGFR-2 (N = 10)** 2	N/A	0.62 [0.37,0.87]	0.56 [0.31,0.80]
*P*-value		0.033	0.029
**Change in SDF1α (N = 9)** 2	N/A	−0.47 [−0.77,−0.17]	−0.56 [−1.04,−0.07]
*P*-value		0.17	0.045

1 Data are shown as Kendall's ***τ*** **β** with approximate 95% confidence intervals between baseline biomarkers and tumor size or outcome measures, with P-value from Kendall's test.

2 Data are shown as Kendall's ***τ*** **β** with approximate 95% confidence intervals between day 28 to baseline ratios of biomarkers and outcome measures, with P-value from Kendall's test.

SD, stable disease; IFP, interstitial fluid pressure; IL-6, interleukin-6; PlGF, placental growth factor; sVEGFR-2, soluble vascular endothelial growth factor receptor-2; SDF1α, stromal cell-derived factor-1-alpha.

### Vascular Density and Maturation and Cancer Cell Proliferation

**Figure 2:**
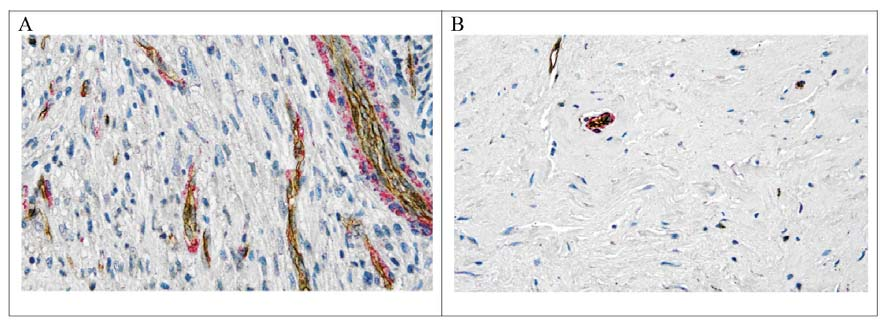
Sorafenib reduces the vessel density in sarcoma lesions. Immunostaining of CD31-positive (brown) or CD31 and α-SMA-positive (brown and pink) tumor vessels before (A) and 28 days after (B) the initiation of sorafenib treatment. Sections were counterstained with hematoxylin. Note the reduced vessel density and cellular content in the sorafenib-treated lesion.

To identify blood vessels and perivascular cells in tumor sections, we performed a double immunostaining procedure with antibodies against CD31 and α-SMA, respectively. In the biopsies of 2 patients, the decrease in vessel density was 59% and 83%, respectively, after sorafenib treatment (**Figure 2** and **Table 5**). The fraction of α-SMA-positive vessels in these 2 patients was 48% and 64%, respectively, before sorafenib treatment, and sorafenib generally reduced the fraction of both α-SMA-negative and -positive vessels (**Table 5**). With sorafenib treatment, there was a trend towards greater reduction in α-SMA-negative than α-SMA-positive vessels (**Table 5**). In a third patient the vessel density was relatively low in the pretreatment biopsies, and increased by approximately 50% after sorafenib (**Table 5**). In 2 patients with sufficient tissue available in both pre- and post-sorafenib biopsies, we also quantified the number of proliferating cancer cells. Sorafenib decreased the percentage of proliferating cancer cells (Ki67-positive) by 27% and 36%, respectively.

**Table 5:** Vascular Density.

Patient	Vessels/field	CD31+/α-SMA+	CD31+/α-SMA−
		vessels per field	vessels per field
Pt#1/Day 0	6.9	3.3	3.6
Pt#1/Day 28	1.2	0.8	0.4
Pt#5/Day 0	15.9	10.1	5.8
Pt#5/Day 56	6.5	4.4	2.1
Pt#13/Day 0	1.2	0.7	0.5
Pt#13/Day 28	1.9	1.7	0.2

### Interstitial Fluid Pressure

IFP measurements were obtained in 6 patients at baseline. The IFP in the 6 lesions varied between 2.5 and 21.0 mm Hg and showed a direct correlation with tumor size (Kendall's tau = 0.87, p = 0.017, **Table 4**). Only 2 of these 6 patients had SD at 28 and 56 days. Thus, corresponding post-sorafenib IFP evaluation was only performed in these 2 patients. In both, a decline in IFP was observed. Tumor IFP decreased from 17.0 to 11.5 mm Hg in one patient and from 3.0 to 0.0 mm Hg in the other. The decrease in tumor IFP in these 2 patients was associated with a reduction in vascular density.

## Discussion

Studying the physiologic and pharmacodynamic impact of mechanistically-targeted drugs is a key aspect of rational therapeutic development and optimization. This study was designed to assess several mechanism-based correlative studies along with standard clinical outcomes. In this cohort of patients with multi-drug refractory STS of varied histologies, sorafenib administration was associated with modest radiographic effects, with a median TTP of 45 days. In a recent study of 145 patients with recurrent or metastatic sarcoma of various histologies treated with sorafenib, RECIST complete or partial responses were observed in five patients with angiosarcoma and one with leiomyosarcoma [30].

While radiographic response criteria have been recently refined [31], they still do not have the sensitivity to detect accurately the more subtle responses which reflect the anti-neoplastic and anti-angiogenic effects of targeted therapies. A set of blood circulating pro-angiogenic and pro-inflammatory molecules are often elevated in patients with tumors and are currently being evaluated as potential biomarkers of response or resistance to treatments such as anti-VEGF therapy [24]. Consistent with the anti-VEGF activity of sorafenib––and in agreement with data from trials in hepatocellular carcinoma patients of another anti-VEGFR TKI sunitinib––treatment increased the plasma concentration of VEGF and PlGF, decreased sVEGFR-2, and increased erythropoiesis [24], [32] [33]. More recently, corroborative data from over 700 patients with renal cell carcinoma in a phase III placebo-controlled randomized trial of sorafenib confirmed that sorafenib therapy increased VEGF and decreased sVEGRF-2 levels [34]. Soluble VEGFR-2 concentration has been previously proposed as a “pharmacodynamic biomarker” for agents with anti-VEGFR-2 TKI activity [24]. Indeed, a greater decrease in plasma sVEGFR-2 correlated with better radiographic response and SD in this study.

Interestingly, we also found significant associations between cytokines that may mediate resistance to anti-VEGF therapy and response: a lower baseline plasma PlGF concentration correlated with a better radiographic response after treatment at day 28, whereas an increase in SDF1α by day 28, correlated with a worse radiographic response after treatment at day 28. The risk of false positive correlations is high given the multiple comparisons and the small sample size. However, it is notable that the same correlations have been seen with other anti-VEGF agents in patients with brain, rectal, and liver cancer (for plasma SDF1α), and in patients with brain, rectal and ovarian cancer (for plasma PlGF) [32], [35], [36], [37], [38], [39].

The sorafenib-induced stabilization of tumor growth in human carcinoma xenografts in mice is associated with a decrease in vascular density [4], [40], [41]. Similarly in two sarcoma patients with stable disease, we found that sorafenib reduced tumor vessel density and IFP. These findings are consistent with sorafenib inhibition of VEGF signaling. We have previously shown that VEGF inhibition by bevacizumab significantly reduces the vascular density and IFP in rectal carcinoma patients [19]. Because VEGF signaling inhibition also reduces the leakiness of tumor vessels, the decrease in IFP may be caused by a reduction in vascular permeability [20]. Sorafenib inhibition of PDGF signaling could also lead to a reduction in IFP.

### Conclusion

Sorafenib shows modest clinical activity in patients with advanced refractory STS. Biomarker changes were consistent with inhibition of angiogenesis by sorafenib, including a mechanism-based decrease in the baseline high levels of intra-tumoral IFP. Preliminary circulating biomarker data from this study suggest a potential biomarker value for sVEGFR-2, PlGF, and SDF1α. Tumor IFP and vessel density appear to decrease when response is maintained. The findings of this hypothesis-generating study should be validated in large prospective trials of sorafenib, alone or in combination with other agents, in sarcoma and other cancers.

## Supporting Information

Table S1 **Changes in number of circulating red blood cells and hemoglobin after sorafenib treatment in advanced STS patients (median values with interquartile range; P value from Wilcoxon test, compared to pretreatment values).**
(DOCX)

Checklist S1 **CONSORT Checklist.**
(DOCM)

Protocol S1 **Trial Protocol.**
(PDF)
