# Supplementary material for: Effects of Sorafenib on Intra-Tumoral Interstitial Fluid Pressure and Circulating Biomarkers in Patients with Refractory Sarcomas (NCI Protocol 6948)
Source: PLoS One. 2012 Feb 7;7(2):e26331. doi: 10.1371/journal.pone.0026331 (PMC3274514; doi:10.1371/journal.pone.0026331)
Supplement: Table S1 — Changes in number of circulating red blood cells and hemoglobin after sorafenib treatment in advanced STS patients (median values with interquartile range; P value from Wilcoxon test, compared to pretreatment values). (DOCX) [file pone.0026331.s001.docx]

**Table S1.** Changes in number of circulating red blood cells and hemoglobin after sorafenib treatment in advanced STS patients (median values with interquartile range; P value from Wilcoxon test, compared to pretreatment values).

|  | **Pretreatment** | **Week 1** | **Week 2** | **Week 3** | **Week 4** | **Week 6** | **Week 8** |
| --- | --- | --- | --- | --- | --- | --- | --- |
| **RBCs (th/cmm)** | 4.0 [3.7,4.1]  N=15 | 4.2 [3.8,4.4]  N=15 | 4.3 [4.1,4.5]  N=13 | 4.1 [3.8,4.3]  N=13 | 4.0 [3.7,4.4]  N=14 | 4.0 [3.9,4.3]  N=8 | 4.1 [4.0,4.3]  N=7 |
| *P-*value | N/A | 0.33 | 0.022 | 0.54 | 0.67 | 0.55 | 0.30 |
| **Hgb (mg/ml)** | 12 [11,13]  N=15 | 13 [11,14]  N=15 | 14 [12,14]  N=13 | 13 [11,13]  N=13 | 13 [11,14]  N=14 | 13 [13,13]  N=8 | 13 [12,14]  N=7 |
| *P-*value | N/A | 0.23 | 0.022 | 0.73 | 0.95 | 0.46 | 0.38 |
