# Supplementary material for: Effects of Sorafenib on Intra-Tumoral Interstitial Fluid Pressure and Circulating Biomarkers in Patients with Refractory Sarcomas (NCI Protocol 6948)
Source: PLoS One. 2012 Feb 7;7(2):e26331. doi: 10.1371/journal.pone.0026331 (PMC3274514; doi:10.1371/journal.pone.0026331)
Supplement: Protocol S1 — Trial Protocol. (PDF) [file pone.0026331.s003.pdf]

PROTOCOL **05-033**

Closed to New Accrual

Closure Effective Date: 05/02/08

No new subjects may be enrolled in the study as described above. Any questions regarding this closure should be directed to the study's Principal Investigator

ALERT PAGE

PROTOCOL **05-033**

Only **DFCI** site is open for participation now.

MGH site will join at a later date

Dana-Farber/Harvard Cancer Center Protocol Front Sheet

DFCI PROTOCOL NUMBER: (assigned by OPRS) **05-033**

1. PROTOCOL TITLE, OVERALL PRINCIPAL INVESTIGATOR, VERSION

Title: A Phase II Clinical and Correlative Study of BAY43-9006 (sorafenib) IND 69,896 in Sarcoma

Other Study Number: **NCI 6948**

Overall PI: Jeffrey A. Morgan, MD **Non-DF/HCC PI:** ☐ Yes **Version # / Date: 5/30/07**

2. DF/HCC STUDY CONTACT INFORMATION

Primary Study Contact for Questions: Conor Devine **Email:** **cdevine1@partners.org** **Phone:** 617-632-4294

OVERALL AND SITE RESPONSIBLE INVESTIGATORS: (List only those under DFCI IRB)

**Overall PI:**	Jeffrey A. Morgan, MD	**Phone:**	617-632-6054	**Institution(s):**	DFCI
**Site Responsible PI:**	Sam Sunghyun Yoon, MD	**Phone:**	617-726-6823	**Institution(s):**	MGH

Collaborating Investigators (name/institution)**:** Monica Bertagnolli, M.D., George Demetri, M.D.,Suzanne George, M.D., David Harmon, M.D., Francis Hornicek, M.D., John Ready, M.D., Chandrait Premand Raut, M.D.,Kathleen Polson, APRN, Andrew Wagner, M.D., Ph.D., James Butrynski, M.D., Amy Potter, APRN, Bonnie Dirr, APRN

Additional Study Staff/Contacts (name/institution)**:** Julie Pokela, Conor Devine, Melissa Hohos, RN (all DFCI) Barbara Rattner,MS, RN, Marguerite Parkman, RN, Natasha Isaac (all MGH)

3. DRUG / DEVICE INFORMATION N/A: ☐

Drugs, Biologics, Devices (Include IND/IDE#): BAY43-9006 (IND #69,896) NSC # 724772

Investigational Drug Brochure (IDB) Version Number / Date (on file with OPRS):Version 6.0,A mend 2.0 / 2/17/2006

4. REGULATORY SPONSOR, PROTOCOL PHASE, MODE AND GRANT INFORMATION

Regulatory Sponsor and Name:
- ☐ Consortium:
- ☐ Cooperative Group:
- ☐ DF/HCC Investigator (Overall PI)
- ☐ Foundation:
- ☐ Industry:
- ☒ NCI / NIH: CTEP
- ☐ Other:

Protocol Mode:
- ☐ Biologic
- ☒ Investigational Drug
- ☐ Investigational Device
- ☐ Expanded Access
- ☐ Observation
- ☐ Prevention
- ☐ N/A

Phase:
- ☐ Pilot Study
- ☐ Phase I
- ☐ Phase I/II
- ☒ Phase II
- ☐ Phase II/III
- ☐ Phase III
- ☐ Phase III/IV
- ☐ Phase IV
- ☐ Feasibility
- ☐ Other:
- ☐ N/A

Protocol Involves: (check all that apply)
- ☒ Chemotherapy
- ☐ Data Repository
- ☐ Genetic Studies
- ☐ Gene Transfer
- ☐ Human Material Banking (tissue, blood, serum, etc.)
- ☒ Human Material Collection (tissue, blood, serum, etc.)
- ☐ Immunotherapy
- ☐ Medical Record Review
- ☐ Questionnaires/Surveys/Interviews
- ☐ Radiological Exams
- ☐ Radiation Therapy
- ☒ Surgery
- ☐ Transplant
- ☐ Vaccine
- ☐ Other:

Grant: ☒ **Grant #:** U01: CA 62490 **Grantee** (name/institution)**:** Donald W. Kufe, MD / DFCI

Subject Population: ☐ **Pediatric**: Age ___ Target Accrual: ☒ **Adult**: Age 18+ Target Accrual: 24

Overall Accrual Goal: 30 Will greater than 25% of the overall study accrual be at DF/HCC: ☒ **Yes** ☐ **No**

Dana-Farber/Harvard Cancer Center: (check all that apply)
- Beth Israel Deaconess Medical Center
- ☒ Brigham and Women's Hospital
- ☐ Children's Hospital
- ☒ Dana-Farber Cancer Institute
- ☐ Dana-Farber Cancer Institute at Faulkner Hospital
- ☒ Massachusetts General Hospital

DF/PCC Network Affiliates: (check all that apply)
- ☐ Cape Cod Healthcare (Hyannis, MA; Mashpee, MA)
- ☐ Emerson Hospital (Concord, MA)
- ☐ Lowell General Hospital (Lowell, MA)
- ☐ New Hampshire Oncology-Hematology-P.A. (Hooksett, NH)
- ☐ North Shore Medical Center Cancer Center (Peabody, MA)
- ☐ South Shore Hospital (S. Weymouth, MA)

DF/HCC Multi-Center Protocols: (list institution/location)

DF/PCC Network Affiliates: (list institution/location)

Dana-Farber/Harvard Cancer Center Protocol Front Sheet

DFCI PROTOCOL NUMBER: (assigned by OPRS) 05-033

Protocol Number: 05-033

Approval Date: 09/27/05 (IRB meeting date when protocol/consent approved or conditionally approved)

Activation Date: 05/31/06 (Date when protocol open to patient entry)

Approval signatures are on file in the Office for Human Research Studies, tel. 617-632-3029.

Date Posted	Revised Sections	IRB Approval Date
06/19/06	Front Sheet replaced due to Amendment #6	06/13/06
09/14/06	Consent Form replaced due to Continuing Review # 1	09/07/06
09/15/06	Consent, Protocol & Front Sheet replaced due to Amendment #7	08/08/06
04/10/07	Consent, Protocol and Front Sheet replaced due to Amendment #8	04/06/07
04/26/07	Protocol and Front Sheet replaced due to Amendment #10	04/17/07
06/28/07	Alert Page Added due to Amendment #9	06/21/07
08/29/07	Consent Form, Protocol and Front Sheet replaced due to amendment #11	08/28/07
09/04/07	Consent Form replaced due to Continuing Review #2 (Previous IRB approval date on CF)	08/16/07
03/19/08	Front Sheet replaced due to Amendment # 12	03/19/08
05/05/05	Permanent Closure to New Accrual: No clinical benefit observed- decision to remove all subjects from study and close enrollment (effective date: 05/02/08; reference: Amendment 13)	05/05/05
08/05/08	Study renewal due to Continuing Review #3	08/05/08
07/27/09	Study renewal due to Continuing Review #4	07/20/09
07/14/10	Study renewal/ CF footer replaced due to Continuing Review #5	06/29/10

TITLE: NCI Protocol 6948: A Phase II Clinical and Correlative Study of BAY43-9006 (sorafenib) IND 69,896 in Sarcoma

Version Date: 5/30/07

Coordinating Center: Dana-Farber/Harvard Cancer Center

***Principal Investigator:** Jeffery A. Morgan, M.D.
Dana-Farber Cancer Institute
44 Binney Street
Boston, MA 02115
Telephone: 617 632-6054
Fax: 617 632-3408
jmorgan@partners.org

Co-Investigators:

Brigham & Women's Hospital

Monica Bertagnolli, M.D.
Surgical Oncology, CA 01-CA-116
75 Francis St., Boston, MA 02115
617 732-8910
mmbertagnolli@bics.bwh.Harvard.edu

John Ready, M.D.
Orthopedics, PBB-Admin-A-Main
75 Francis St., Boston, MA 02115
617-732-5368
jready@bics.bwh.Harvard.edu

Chandrait Premand Raut, M.D.
Division of Surgical Oncology
75 Francis St., Boston, MA 02115
craut@partners.org

Dana-Farber Cancer Institute

George Demetri, M.D.
44 Binney Street,
Boston, MA 02115
617 632-3985
Fax: 617 632-3408
gdemetri@partners.org

Suzanne George, M.D.
44 Binney Street,
Boston, MA 02115
617-632-6826
Fax: 617 632-3408
sgeorge2@partners.org

James Butrynski, M.D.
44 Binney Street,
Boston, MA 02115
Telephone: 617 632-5204
Fax: 617 632-3408
jbutrynski@partners.org

Andrew Wagner, MD
44 Binney Street
Boston, MA 02115
Telephone: 617 632-4899
Fax: 617 632-3408
awagner@partners.org

Massachusetts General Hospital
David Harmon, M.D.
Hematology-Oncology, Cox 6
100 Blossom St, Boston, MA 02114
Telephone: 617 726-8748
Fax: 617 724-3166
dharmon@partners.org

Francis Hornicek, M.D.
Surgical Oncology
55 Fruit Street, Gray/Bigelow 6-604c
Boston, MA 02114
Telephone: 617 724-3700
Fax: 617 746-6823
fhornicek@partners.org

Sam Sunghyun Yoon, MD
Surgical Oncology
100 Blossom Street, Cox 626
Boston, MA 02114-2617
Telephone: 617 724-3700
Fax: 617 746-6823
ssyoon@partners.org

Statistician:

David M. Zahrieh
Dana-Farber Cancer Institute
Telephone: 617-632-2455
Fax: 617-632-2444
david_zahrieh@dfci.harvard.edu

Responsible Research Nurses:

Jennifer Harrington, RN
Dana-Farber Cancer Institute
Telephone: 617-632-2201
jennifer_harrington@dfci.harvard.edu

Michael T. Quigley, RN
Dana-Farber Cancer Institute
Telephone: 617-632-5117
michael_quigley@dfci.harvard.edu

Responsible Data Manager:

Jeanine Salesi
DFCI
617-632-5912
Jeanine_Salesi@dfci.harvard.edu

NCI Supplied Agent: BAY 43-9006 (sorafenib) NSC#724772, IND # 69,896

SCHEMA

A Phase II Clinical and Correlative Study of BAY43-9006 (sorafenib) IND 69,896 in Sarcoma

→ Register patient
→ Interstitial Fluid Pressure, biopsy, functional imaging
→ administer BAY 43-9006 orally bid for 14-(Treatment Group I) or 28 (Treatment Group II)-day cycle
→ repeat baseline studies at day 14 or 28
→ discontinue upon complete resection (Treatment Group I) or evidence of disease progression or unacceptable toxicity (Treatment Group II).

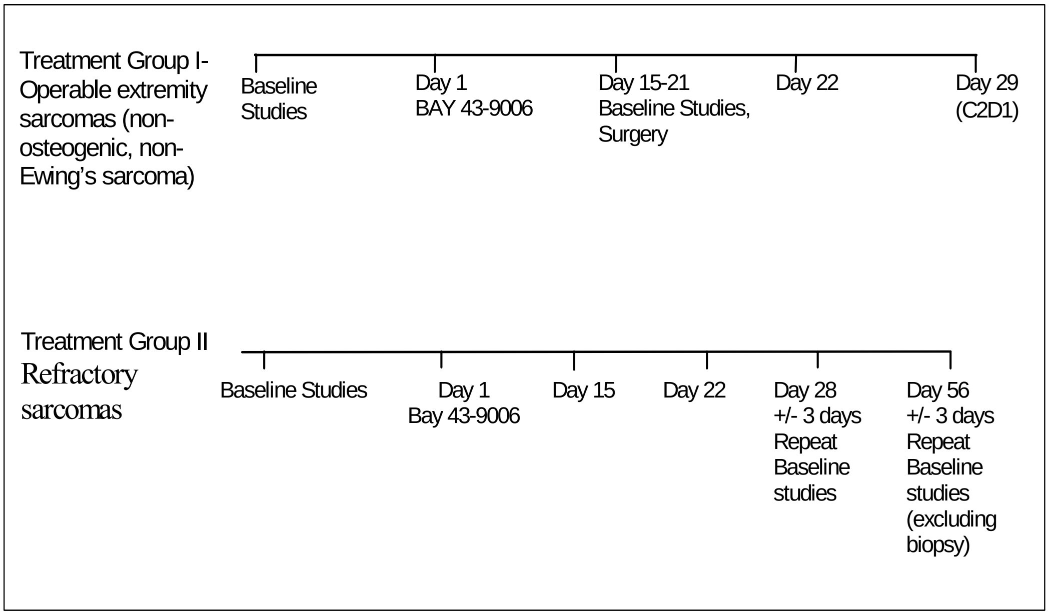

Eligibility Criteria

- Bone and soft tissue sarcomas other than localized osteosarcoma and Ewing's sarcoma, either resectable or unresectable and for which there is no known curative or standard palliative chemotherapy regimen.
- Tumor amenable to serial biopsy and measurement of interstitial fluid pressure without excess risk to the patient.
- Age ≥ 18 years.
- ≥ 3 weeks since chemotherapy or radiation therapy (6 weeks for nitrosoureas or mitomycin-C), if applicable.
- ≥ 3 weeks since major surgery
- ECOG performance status ≤ 2.
- Life expectancy ≥ 2 months.
- No uncontrolled serious medical or psychiatric illness.
- No active brain metastases
- Patients who are on warfarin anticoagulation are allowed to participate as long as they are converted to a low molecular weight heparin (e.g. lovenox) from study entry until at least day 56.
- Women of childbearing potential must not be pregnant or lactating.
- Fertile males and females must use adequate contraception.
- Signed informed consent.

Laboratory Values

ANC	≥ 1,500/mm^3
Platelets	≥ 100,000/ mm^3
SGOT	≤ 2.5-times the upper limit of normal
SGPT	≤ 2.5-times the upper limit of normal
Total bilirubin	≤ upper limit of normal
Serum creatinine	≤ 1.5 times the upper limit of normal

On-study Evaluations

- Biopsy for tissue diagnosis (if not previously obtained), biopsy for correlative studies, digital contrast enhanced MRI (dce-MRI) or functional CT and interstitial fluid pressure measurement.
- Pharmacokinetics and pharmacodynamic sampling: cycle 1
- Toxicity assessment: continuous
- Hematology and serum chemistries: once weekly.
- Follow-up upon discontinuation: One month telephone followup for all study-related toxicity

ABSTRACT

In many tumors, interstitial fluid pressure (IFP) is elevated uniformly throughout each tumor, with a sharp gradient only apparent at the periphery. The causes are still under investigation but are thought to include impaired lymphatic drainage and markedly increased vessel/ endothelial cell (EC) permeability, or compression of capillaries and lymphatic vessels from tumor growth in a confined space. The increased accumulation of fluid produces elevated IFP with a further decrease in perfusion of tumors alongside a resultant decrease in tissue oxygenation and drug delivery, especially delivery of macromolecules. IFP may occur in many tumor types and may be an independent predictive factor for survival in cervical cancer

In patients with extremity sarcomas, increased tumor IFP has several clinically important implications. Increased tumor pressure produces pain. The pain results in decreased mobility. When combined with venous compression and reduced venous return, this increases the incidence of venous thromboembolism before and immediately after surgery with consequent morbidity and risk of mortality. The IFP increases the risk of tumor cell spillage and extravasation at operation.

Two proteins and their tyrosine kinase receptors on the EC, VEGF/VEGFR-2 and PDGF/PDGFβ mediate this increase in permeability. Preclinical studies have shown that antagonists to each protein/receptor can increase the uptake and effect of anticancer agents. Clinical trials of the monoclonal antibody bevacizumab (Avastin™) in combination with irinotecan/5-fluorouracil/leucovorin (IFL) have shown a significant survival advantage in metastatic colorectal cancer compared to IFL alone and certainly compared to bevacizumab alone.

BAY43-9006/sorafenib is a small molecule tyrosine kinase inhibitor of *c-raf/b-raf* and VEGFR-2 and PDGFRβ, with micromolar IC_{50} to the purified receptors. BAY 43-9006 has a phase II dosing regimen of 400 mg bid p.o. It has single agent activity against renal cell carcinoma and hepatocellular carcinoma and can be combined with many chemotherapy agents at or near full dose, including doxorubicin at 60 mg/m^2 (Investigational Drug Brochure, Bayer, 2003). As a multi-targeted tyrosine kinase inhibitor, the effects of BA43-9006 on both the VEGRR-2 and PDGFRβ pathways should antagonize the vascular/EC permeability that initiates/contributes to increased IFP, resulting in decreased pressure within the tumor as well as decreased pain and venous compression, increased blood flow and, in the case of concomitant chemotherapy, increased drug delivery and enhanced antineoplastic effect.

TABLE OF CONTENTS

SCHEMA 2
ABSTRACT 5
TABLE OF CONTENTS 6
1.0 Introduction 8
1.1 Elevated interstitial fluid pressure in cancer 8
1.2 BAY 43-9006/SORAFENIB, A COMBINED VEGFR-2 AND PDGFββ INHIBITOR 9
    1.2.1 Mechanism of Action of BAY 43-9006 9
    1.2.2 *In Vivo* Antitumor Activity 10
    1.2.3 Preclinical Toxicology 11
1.3 Preclinical Pharmacokinetics and Drug Metabolism 12
1.4 Clinical Studies of BAY 43-9006 12
    1.4.1 Introduction 12
    1.4.2 Clinical Pharmacology 12
    1.4.3 Summary of Relationship between pharmacokinetics, safety, and efficacy 14
    1.4.4 Clinical Efficacy Data 14
    1.4.5 Clinical Toxicity 14
1.5 Recommended Phase II Dose 16
2.0 Correlative Studies of Elevated IFP 17
2.1 Rationale 17
2.2 Angiogenesis and Biological/Physiological Marker 17
    2.2.1 Perfusion CT scans have been employed in assessing responses of soft tissue sarcomas and/or other malignancies to radiation therapy and/or chemotherapy 17
    2.2.2 Targeting angiogenic vessels requires adequate methods for the assessment of the biologic effect of various new drugs developed to control cancer progression 18
    2.2.3 The number of circulating endothelial cells (CECs), measured by flow cytometry, is significantly increased in the peripheral blood of untreated lymphoma and breast cancer patients. 18
    2.2.4 The strong correlation observed between the number of CECs and tumor volume indicates that CECs increase with tumor progression 18
    2.2.5 In conclusion, CEC evaluation is a relevant surrogate, non-invasive angiogenesis marker that may contribute to the existing panel of angiogenesis assays 18
    2.2.6. Studies from our group and other investigators have shown that the interstitial fluid pressure (IFP) in colon, breast, lung, head and neck, cervix and skin tumors is significantly higher than in normal tissues 18
2.3 Summary for study of sorafenib/BAY 43-9006 and Correlative Biology 19
3.0 Objectives 19
3.1 Primary Objectives 19
3.2 Secondary Objectives 20
4.0. Patient Selection 20
4.1 Eligibility Criteria 20
4.2 Exclusion Criteria 21
5.0 Registration 22
6.0 Drug Treatment plan 22
6.1 Pharmaceutical information 22
    6.1.1 How Supplied 23

6.1.2 Reported Adverse Events and Potential Risks 23
6.1.3 Method of Administration 23
6.1.4 Potential Drug Interactions 23
6.1.5 Bay 43-9006 Availability, Ordering and Accountability 24
6.2 Pretreatment Evaluations 24
6.3 On-Study Evaluations 24
6.4 Drug Administration and Treatment Plan 25
6.5 Patient Re-treatment 27
6.6 Dose Modifications 27
6.7 Duration of Treatment 27
6.8 Withdrawal from Therapy 28
6.9 Concurrent Supportive Care 28
6.10 Toxicity 29
6.10.1 Evaluations 29

6.11 Adverse Event Reporting Requirements 30
7.0 Correlative Studies 35
7.1 Schedule of Correlative Studies Before, During and After BAY 43-9006 initiation 35
7.2 Methods 35
7.2.1 Functional Imaging 35
7.3 Immunohistochemistry 36
7.4 IFP Measurements 36
7.5 Circulating cells and VEGF measurements 37
7.6 ELISA assays 37
8.0 Surgery 37
9.0 DEFINITIONS OF DISEASE RESPONSE 37
9.1 Definitions 37
9.2 Guidelines for Evaluation of Measurable Disease 38
9.3 Response Criteria 39
10.0 Schedule of Assessments 42
11.0 Statistical Considerations 43
12.0 Pharmacokinetic Studies 44
13.0 Monitoring and Quality Asurance 46
13.1 Informed Consent 46
13.2 Institutional Reviews 46
13.3 Publication of Data 46
13.4 Inclusion of Women and Minorities 46
14.0 Cooperative Research and Development Agreement (CRADA) / Clinical Trials Agreement (CTA) 46
15.0 References 48

1.0 Introduction

1.1 Elevated interstitial fluid pressure in cancer

Chemotherapy is a major treatment modality for solid tumors. Improving the distribution of drugs between normal tissues and tumors may potentially reduce toxic side effects and to achieve higher efficacy of chemotherapeutic drugs. One property of most solid tumors that has been suggested as a potential target for efforts to increase tumor drug uptake is tumor interstitial hypertension.[1,2] Increased tumor interstitial fluid pressure (IFP) acts as a barrier for tumor transvascular transport.[3,4] Reduction of tumor IFP, or modulation of microvascular pressure, has been shown to increase transvascular transport of tumor-targeting antibodies or low molecular weight tracer compounds.[5,6,7,8]

The etiology of interstitial hypertension in tumors is poorly understood. The scarcity of lymphatic vessels in tumors has been proposed as one factor contributing to the increased tumor IFP.[2] The microvasculature and the supporting stroma compartment are also likely to be important determinants for tumor IFP.[9,10] Growing evidence indicates that the transmembrane platelet derived growth factor receptor β (PDGFRβ) tyrosine kinase as an interesting candidate target for pharmacological intervention of tumor interstitial hypertension.[7,11] A role for PDGFβ in anaphylaxis has been proposed, where PDGFRβ stimulation was found to normalize the dextran-induced lowering of the IFP.[11] In contrast, activation of the structurally related PDGFR α had no effect on loose connective tissue IFP. A role for PDGFRβ that control tumor IFP was demonstrated in a syngeneic rat colon adenocarcinoma model.[7] In this tumor model (PROb), with PDGFRβ expression restricted to tumor stromal cells, a significant reduction in tumor IFP was observed after treatment with a DNA aptamer that inhibits the PDGFRβ ligands PDGFαβ and -ββ. Finally, the well-documented PDGFRβ expression in the stromal compartment in many common solid tumors, *e.g.* lung, breast, and colon carcinoma, which are also characterized by tumor interstitial hypertension, is consistent with a role for PDGFRβ in the controlling of tumor IFP.[12]

Pietras investigated the effects of PDGFR inhibition on the efficacy of two commonly used cytotoxic drugs, paclitaxel and 5-FU, in tumor models with PDGFR expression restricted to the tumor stroma. Inhibition of PDGF receptors alters tumor uptake of chemotherapeutical agents. For PDGFR inhibition they used a PDGFβ specific aptamer and imatinib, a low molecular weight tyrosine kinase inhibitor, which selectively blocks the PDGF receptor kinase, the *c-ki*t receptor kinase, and the *abl* and *arg* nonreceptor kinase.[9] The study demonstrated that PDGF receptor inhibition in tumor stromal cells reduces tumor IFP, increases the tumor uptake of chemotherapy drugs, and enhances their therapeutic effects. The study thus identifies inhibition of PDGF receptor signaling in tumor stroma as a novel, possibly general, combination strategy for enhancement of the therapeutic effects of standard chemotherapeutics.

Vascular endothelial growth factor (VEGF), also known as vascular permeability factor, plays a crucial role in developmental, physiologic, and pathologic angiogenesis. Willet at al have shown that the VEGF-specific antibody bevacizumab significantly reduces IFP in colorectal cancer patients. This decrease may result from the “normalization” of function within the tumor vasculature, including the re-appearance of vascular smooth muscle cells.[13] There is a decrease in vascular volume and microvessel density within the tumor, along with a reduction in the vascular–permeability surface and extravasation, which results in decreased IFP and allows greater penetration of small molecules, including iodinated contrast dyes. Clinical trials of

bevacizumab (Avastin™) in combination with irinotecan/5-fluorouracil/leucovorin (IFL) have shown a significant survival advantage in metastatic colorectal cancer compared to IFL alone and certainly compared to bevacizumab alone.[10, 11] This may be due to increased drug delivery, but that relationship remains to be proved.

1.2 BAY 43-9006/SORAFENIB, A COMBINED VEGFR-2 AND PDGFββ INHIBITOR

BAY43-9006/sorafenib is a small molecule tyrosine kinase inhibitor of *c-RAF/B-RAF* and VEGFR-2 and PDGFRβ, with micromolar IC_{50} to the purified receptors. BAY 43-9006 has a phase II dosing regimen of 400 mg bid p.o. It has single agent activity against renal cell carcinoma and hepatocellular carcinoma and can be combined with many chemotherapy agents at or near full dose, including doxorubicin at 60 mg/m^2 and the combination of paclitaxel/carboplatin (Investigational Drug Brochure, Bayer, 2003). As a multi-targeted tyrosine kinase inhibitor, the effects of BAY43-9006 on both the VEGFR-2 and PDGFR-β pathways should antagonize the vascular/EC permeability which initiates/contributes to increased IFP and result in decreased pressure within the tumor with decreased pain and venous compression, increased blood flow and, in the case of concomitant chemotherapy, increased drug delivery and enhanced antineoplastic effect.

1.2.1 Mechanism of Action of BAY43-9006

BAY 43-9006 was selected based on the inhibition of the enzyme, *raf* kinase, in a battery of biochemical, cellular, and *in vivo* assays. The *ras/raf* signaling pathway is an important mediator of responses to growth signals and angiogenic factors. This pathway is often aberrantly activated in human tumors due to presence of activated *ras*, mutant *b-raf*, or overexpression of growth factor receptors. Therefore, inhibition of the *raf*/MEK/ERK, mitogen-activated kinase (MAPK) signaling pathway in tumors may be of clinical benefit. The novel bi-aryl urea, BAY 43-9006, is a potent inhibitor of *c-raf* and wild-type and mutant (V599E) *b-raf in vitro* with IC50s of 2 nM, 22 nM and 38 nM, respectively. Further characterization of BAY 43-9006 revealed that this novel agent also inhibits several receptor tyrosine kinases (RTKs) that are involved in tumor progression (human (h)VEGFR-2, murine (m)VEGFR-2, mVEGFR-3, mPDGFR-β, Flt3, and c-KIT) along with p38α , a member of the MAPK family. In cellular mechanistic assays, BAY 43-9006 reduced basal phosphorylation of the MAPK pathway in a panel of human breast, melanoma, pancreatic, and colon tumor cell lines. In other cellular assays, BAY 43-9006 was found to be a potent inhibitor of human and mVEGFR-2, mVEGFR-3, and mPDGFR-β receptor phosphorylation.

Oral treatment once daily with BAY 43-9006 has demonstrated broad-spectrum antitumor efficacy in preclinical tumor xenograft models in athymic mice. Immunohistochemical or Western staining of human tumor xenografts *ex vivo* with a phospho-specific polyclonal anti-ERK1/2 antibody demonstrated inhibition of the MAPK pathway after 5 days of treatment with BAY 43-9006 in 4/6 tumors examined (HT-29, DLD-1, HCT-116, and MDA-MB-231 but not in Colo-205 or Mia-PaCa-2). In addition, in 2/2 human tumor xenografts (MDA-MB-231 and Colo-205) that were stained for CD31 expression from the same tumor samples used for the pERK studies, there was a dramatic reduction of tumor neovascularization. These data suggest that BAY 43-9006 may inhibit tumor progression through multiple mechanisms by inhibiting tumor

cell proliferation that is dependent on activation of the MAPK pathway and by inhibiting tumor angiogenesis or neo-vascularization through inhibition of VEGFR-2, VEGFR3, and/or PDGFR-β. In addition, recent data also indicate that inhibition of *c-raf* may promote cell death in endothelial cells as a downstream event of VEGFR-2 stimulation*.

**Summary of the In vitro Profile of BAY 43-9006**	
**Biochemical Assay a IC50 (μM)**	
c-raf b	0.002/0.006
b-raf wild-type	0.025
b-raf V599E mutant	0.038
VEGFR2	0.090
mVEGFR2	0.006
mVEGR3	0.010
mPDGFRβ	0.028
Flt3	0.058
c-KIT	0.068
FGFR1	0.580
p38α	0.038
***Cellular Mechanism c* IC50 (μM)**	
MDA MB 231 MEK phosphorylation (Human Breast)	0.04
BxPC-3 MEK phosphorylation (Human Pancreatic)	1.00
LOX ERK phosphorylation (Human Melanoma)	0.80
b-raf ER MEK activation (Human Chimera, 3T3 cells)	2.30
VEGFR2 phosphorylation (Human, 3T3 cells)	0.03
VEGFR3 phosphorylation (Mouse, 293 cells)	0.10
PDGFRβ☐ phosphorylation (Human AoSMC)d	0.02

1.2.2 *In Vivo* Antitumor Activity

BAY 43-9006 demonstrated *in vivo* antitumor efficacy against a broad range of human tumor xenografts as summarized in Figure 4-1. In this figure, BAY 43-9006 was administered p.o. once a day for 9 days against each tumor model shown. Additional studies are pending in xenograft models of renal cell carcinoma and melanoma. As shown in Figure 4.1 from the IDB, BAY 43-9006 exhibits antitumor efficacy against tumor models that express either mutated *k-ras* (DLD-1, Mia-PaCa-2) or *braf* (Colo-205, HT-29). The compound is also effective against the MDA-MB-231 model that exhibits mutation of both genes. BAY 43-9006 was also effective against the SK-OV-3 human ovarian tumor line that contains a wild-type *ras* and *b-raf* but overexpresses both the EGF and Her2 growth factor receptors. These receptors also signal through the *ras/raf*/MEK pathway. The efficacy of BAY 43-9006 against this tumor at 14 days after tumor implantation model suggests that a *raf* kinase inhibitor may have utility not only in human tumors containing *ras* and/or *b-raf* mutations, but also in tumors that overexpress other growth factor receptors that signal through the same pathway.

.

Figure 4-1: Antitumor efficacy of BAY 43-9006 against human tumor xenografts

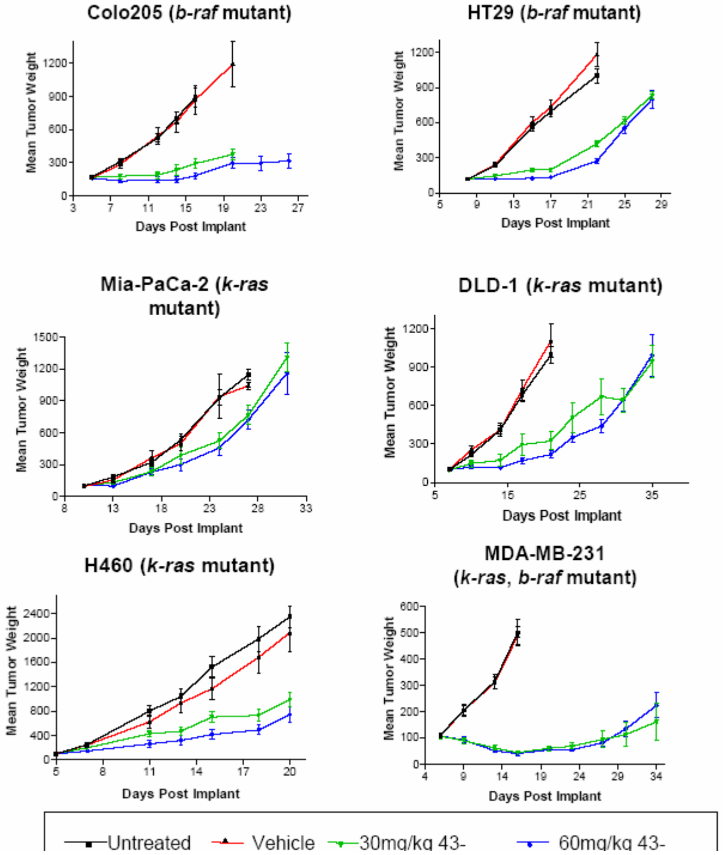

1.2.3 Preclinical Toxicology

The preclinical toxicity profile of BAY 43-9006 can be summarized as follows. Short-term high-dose treatment was well tolerated clinically by rats, mice and dogs; a plateau in systemic exposure was observed. Exposure-dependent mortality (mean time to death ≥ 3 weeks) occurred without preceding specific signs of morbidity in rats; dose-limiting toxicity in dogs was gastrointestinal (emesis, bloody diarrhea). The lowering of threshold-dose for significant toxicities occurred with duration of exposure. Histopathology revealed degeneration/regeneration processes in multiple organ systems including liver, kidneys, lymphoreticular/ hematopoietic system, GI tract, pancreas, adrenals, reproductive organs, skin, teeth, and bone. No main target organ of toxicity could be identified; some morphological lesions were not reversible within 4 weeks (e.g., bile duct proliferation, liver fibrosis, adrenal necrosis, effects on lymphoreticular system) following a 4 week course of treatment. Changes in hematology and clinical chemistry were poor predictive indicators for specific organ lesions. Serum levels of hepatic transaminases were markedly, but not consistently, elevated. At maximum tolerated doses, in terms of lethality in 3-month studies, systemic exposure at steady state was comparable in rats and dogs (AUC_{0-24} about 35 mg·h/l), but significantly higher in mice (AUC_{0-24} about 147 mg·h/l). Based on genotoxicity assays conducted, BAY 43-9006 does not exhibit a significant risk of genotoxicity to patients. There is potential to adversely affect fertility and reproductive performance, based on

repeated-dose toxicity studies in rats. Preliminary data suggest an affect on embryonic and fetal development.

1.3 Preclinical Pharmacokinetics and Drug Metabolism

Absorption in mice, rats and dogs ranged from 69% in dogs to 92% in mice and bioavailability in mouse and rats was ~79% and in dogs was ~61%. In mice, rats and dogs the plasma clearance was low (CL<0.21 l/h/kg) and the Vss was low (~0.7 – 0.9 l/kg). Excretion of the compound is primarily via the biliary/fecal route in rats and dogs. Protein binding was high and was species dependent (mean free fraction expressed as %) 2.5% in mouse, 1.6% in dog, 1.4% in rat, 1.2% in human. BAY 43-9006 showed a market potency to inhibit several CYP isoforms involved in drug metabolism and clinically relevant drug-drug interactions with substrates of different isoforms appear to be possible. In vitro model of enzyme induction using human hepatocyte showed no significant induction of CYP 1A, 2C9, 2C19 and 3A enzymes. CYP 3A4 is the decisive enzyme responsible for the metabolism of BAY 43-9006 in vitro in man

1.4 Clinical Studies of BAY 43-9006

1.4.1 Introduction

BAY 43-9006 has been evaluated in multiple Phase I and Phase II studies in a variety of advanced tumor types. To date, over 500 patients have been treated with single-agent BAY 43-9006. In general, BAY 43-9006 was safely administered chronically in most patients. The majority of patients experienced adverse events at some time during the treatment with BAY 43-9006. Most of these events were not severe and were manageable. Unlike cytotoxic agents, which usually cause hematologic effects such as thrombocytopenia, neutropenia, and anemia, BAY 43-9006 generally did not exhibit this property. Typically, patients experienced skin-related symptoms either hand-foot syndrome and/or a rash. Fatigue and diarrhea were also common but usually low to moderate grade. These toxicities resolved once the drug was discontinued. Antitumor activity was seen in both Phase I and II studies in multiple tumor types including renal cell cancer (RCC). Advanced RCC is a fatal condition with limited therapeutic options. Due to the recent positive results seen in RCC, Bayer has made a decision to pursue further development of BAY 43-9006 in Phase III trials.

1.4.2 Clinical Pharmacology

Key Points:

- There is a less than proportional increase in C_{max} and AUC values with increasing dose.
- Moderate-fat meal does not affect BAY 43-9006 bioavailability.
- Consistent with its half-life, plasma BAY 43-9006 accumulates upon multiple dosing.
- There is no further increase in BAY 43-9006 C_{max} and/or AUC values beyond 7 days of multiple dosing.
- BAY 43-9006 generally accounts for approximately 64-86% of the circulating analytes after at least 7 days of multiple dosing.
- Approximately 19% of the dose is excreted in urine and 76% in feces. BAY 68-3472 is the major metabolite in plasma.

- BAY 43-9006 exhibits high interpatient pharmacokinetic variability that is not explained by age, race, gender and body weight. At a given dose, BAY 43-9006 exposure does not appear to correlate with clinical toxicity. The relationship between dose and anti-tumor activity cannot reliably be estimated on the basis available data.
- The dose of 400 mg bid exhibits a similar safety profile in hepatoma patients with either Child's Pugh A or Child's Pugh B hepatic function status even though preliminary data show numerical differences in PK.
- In vitro liver microsomal data indicate that BAY 43-9006 inhibits a number of hepatic cytochrome P450 isoenzymes. The following Ki-values were obtained: CYP2B6: 6.2 µM, CYP2C8: 2.4 µM, CYP2C9: 7.3 µM, CYP2C19: 17 µM, CYP2D6: 4.2 µM, and CYP3A4: 4.9 µM. BAY 43-9006 is not considered to be a mechanism-based inhibitor. Additionally, in vitro metabolism data indicate that BAY 43-9006 is primarily metabolized by CYP3A. The possible effect that inhibitors of CYP 3A may have on BAY 43-9006 is unknown.

The following Table from the Investigational Drug Brochure summarizes the pooled data from the Phase I trials

Table 5-1: Mean Plasma BAY 43-9006 Pharmacokinetic Parameters following at least 7 Days Dosing in Cancer Patients (Studies 100283, 10164, 100277 and 100342).

Dose		AUC (0-12) [mg*h/L]	C_{max} [mg/L]	T_{max} [h]	$T_{1/2}$ [h]
100 mg bid	N=	18	19	18	10
	Geo Mean/ median	32.4	4.10	2.0	31.6
	Approx CV% / range	54	58	[0-12]	51
200 mg bid	N=	19	20	18	9
	Geo Mean/ median	35.6	4.5	3.0	26.9
	Approx CV% / range	68	67	[0-24.1]	24
300 mg bid	N=	9	9	9	3
	Geo Mean / median	41.7	5.1	3.2	29.5
	Approx CV% / range	58	76	[2-6]	11
400 mg bid	N=	39	41	31	10
	Geo Mean / median	57.3	7.1	3.1	24.4
	Approx CV% / range	59	60	[0.5-24.3]	29
600 mg bid	N=	37	43	34	16
	Geo Mean / median	73.2	8.8	3.0	29.5
	Approx CV% / range	57	54	[0.08-24.3]	35
800 mg bid	N=	10	12	11	4
	Geo Mean / median	72.1	9.5	2.1	36.0
	Approx CV% / range	41	34	[0-24]	36

Note: AUC(0-12) is the AUC value, over the dosing interval, following at least 7 days of dosing, over a nominal period of 12 hours. The exact time interval may be different for each patient. Geo Mean = Geometric mean; Approx CV% = Approximate coefficient of variation. Median, instead of geometric mean, and range instead of approximate CV% are presented for t_{max}.

.

Data from four ascending dose studies (Table 5-1) show that BAY 43-9006 exhibits high interpatient pharmacokinetic variability. There is a less than proportional increase in BAY 43-9006 C_{max} and AUC values with increasing doses from 100 mg bid to 800 mg bid. Figure 5-2 depicts the high interpatient pharmacokinetic variability and the less than proportional increase in mean $AUC_{(0-12)}$ values with increasing dose. In order to achieve higher BAY 43-9006 exposures, different dosing regimens were evaluated. Clinical pharmacokinetic studies were conducted early in Study 100283 to evaluate the impact of dosing BAY 43-9006 once daily vs. twice daily. A few patients received two treatments in a cross-over fashion: 200 mg BAY 43-9006 once daily and 100 mg BAY 43-9006 twice daily. The total daily dose was 200 mg in both treatments. BAY 43-9006 $AUC_{(0-\infty)}$ values were, on an average, 57% greater following administration of 100 mg twice daily compared to the administration of 200 mg once daily. BAY 43-9006 has a long half-life and accumulates 4-6 fold upon multiple dosing. The evaluation conducted here indicated that, despite significant accumulation upon multiple dosing, higher exposure could be achieved following twice daily administration compared to once daily administration. Since administration of the same total daily dose (200 mg) twice-daily resulted in a 57% higher AUC compared with once-daily, a decision was made to use the twice daily (bid) dosing regimen for all future clinical evaluations. Multiple dose accumulation of BAY 43-9006 has been evaluated in all ongoing Phase I clinical trials. There was up to 15-fold accumulation of BAY 43-9006 estimated from the ratio of steady state $AUC_{(0-12)}$ to Day 1 $AUC_{(0-12)}$ values. There is considerable interpatient variability in BAY 43-9006 accumulation upon multiple dosing. Comparison of Day 7 and Day 21 exposure data in Study 10164 showed that there was no further increase in BAY 43-9006 AUC values after 7 days of dosing. The effect of a moderate-fat breakfast and a high-fat breakfast on BAY 43-9006 bioavailability has been evaluated in 15 healthy volunteers. There was no clinically relevant effect of a moderate-fat meal (14% increase in AUC and 7% decrease in C_{max}) on BAY 43-9006 bioavailability. However, a high fat meal caused at 29% decrease in BAY 43-9006 AUC and a 38% decrease in BAY 43-9006 C_{max} values.

1.4.3 Summary of Relationship between pharmacokinetics, safety, and efficacy

Plasma BAY 43-9006 C_{max} and AUC values have been evaluated against anti-tumor activity. At a given dose, there was no apparent relationship between C_{max} and/or AUC and anti-tumor response. This may be because of limited anti-tumor activity data in Phase I trials in which pharmacokinetics was evaluated. Plasma BAY 43-9006 AUC values have been evaluated against study drug-related Grade 3 and 4 toxicities. There is no apparent relationship between Grade 3 and 4 toxicities and exposure at a given dose. BAY 43- 9006 AUC values have also been evaluated against skin-related toxicities and diarrhea. At a given dose, there is no apparent relationship between C_{max} and/or AUC and skin-related toxicities and diarrhea.

1.4.4 Clinical Efficacy Data

The Phase I studies were not designed to evaluate antitumor activity of BAY 43-9006 as a primary endpoint. Antitumor efficacy was evaluated as a secondary endpoint using modified WHO tumor response criteria or the RECIST criteria. Objective tumor shrinkage has been documented and confirmed in 2 patients. Patient 10-1 in Study 10283 is a 26 year-old male with HCC with pelvic mass who experienced a partial response (RECIST criteria) at a BAY 43-9006 dose of 400 mg bid continuously. Patient 01-22 on Study 10164 is a 54 year old male with RCC

with lung metastasis who experienced a partial response (WHO criteria) at a dose of 600 mg bid (21 days on 7 days off). The responses in these phase I trials is summarized in the following Table from the IDB:S

Table 5-4: Summary statistics for response data by initial dose – population: all subjects valid for safety

			100 bid (N=18)	200 bid (N=18)	400 bid (N=31)	600 bid (N=38)	800 bid (N=9)
Best response	Missing	N (%)	1(6)	2 (11)	3(10)	7(18)	3(33)
	PR	N (%)			1(3)	1(3)	
	MR	N (%)	1(6)		1(3)	1(3)	
	SD	N (%)	5(28)	7(39)	11(35)	16(42)	2(22)
	PD	N (%)	11(61)	9(50)	15(48)	13(34)	4(44)

PR, SD, PD were reported by investigator

PR: Partial response defined as > 30% decrease by RECIST criteria and > 50% by WHO criteria

MR: Tumor shrinkage is between 25-49% for at least 2 consecutive scans if WHO criteria was used and tumor shrinkage of 15 - 29% for at least 2 consecutive scans if RECIST criteria was used

SD: Steady state of disease neither sufficient shrinkage to qualify for PR and MR nor sufficient increase to qualify for PD by RECIST criteria and < 50% decrease and < 25% increase by WHO criteria

PD: 20% increase by RECIST criteria and > 25% increase by WHO criteria

Preliminary data in the Phase II randomized discontinuation study show at the 12-week assessment period, 18 patients with advanced RCC were evaluable for response. These patients who failed prior anticancer therapy were treated with a daily dose of BAY 43-9006, 400 mg bid. Of these 18 patients, as per investigator assessment, 7 patients had minor regressions (did not meet formal response criteria), but as per protocol had greater than 25% reduction in tumor size per the modified WHO. Of 10 patients with soft tissue sarcoma, there were 3 unconfirmed partial responses.

1.4.5 Clinical Toxicity

Over 400 patients have been treated with BAY43-9006 in phase I trials with a variety of schedules. Toxicity was associated with increased dose, as was expected. Table 5.6 from the IDB details the AE seen in the bid dosing schedule proposed for this trial.

Table 5-6: Incidence of treatment-emergent adverse events causing dose reduction (worst grade) in Phase I

Adverse Event/ NCI Grade	100 bid (N=18)	200 bid (N=18)	400 bid (N=31)	600 bid (N=38)	800 bid (N=9)
Any AE					
1	0(0.0%)	0(0.0%)	0(0.0%)	2(5.3%)	0(0.0%)
2	1(5.6%)	3(16.7%)	2(6.5%)	3(7.9%)	3(33.3%)
3	1(5.6%)	1(5.6%)	2(6.5%)	4(10.5%)	1(11.1%)
4	0(0.0%)	0(0.0%)	0(0.0%)	1(2.6%)	0(0.0%)
All	2(11.1%)	4(22.2%)	4(12.9%)	10(26.3%)	4(44.4%)

Since all patients in this trial will receive 400 mg bid, the adverse events in the phase II trial in the randomized discontinuation/RCC may more accurately reflect the risks to patients in this trial (Table 5.9). Since this trial is ongoing, a final audit has not been completed and these events are those reported by the investigators.

Table 5-9: Incidence (percentage) of selected* treatment-emergent adverse events in 100391 (Phase II) per worst grade (drug related) (N=234)

Adverse Event/ NCI Grade	Grade 1	Grade 2	Grade 3	Grade 4	All
Hand-foot skin reaction	34(14.5%)	16(6.8%)	16(6.8%)	0(0%)	66(28.2%)
Dermatology/skin-others	56(23.9%)	6(2.6%)	0(0%)	0(0%)	62(26.5%)
Alopecia	38(16.3%)	2(0.8%)	0(0%)	0(0%)	40(17.1%)
Rash/desquamation	37(15.8%)	9(3.8%)	8(3.4%)	0(0%)	54(23.1%)
Fatigue (lethargy, malaise, asthenia)	48(20.5%)	17(7.3%)	6(2.6%)	0(0%)	71(30.4%)
Anorexia	27(11.5%)	6(2.6%)	1(0.4%)	0(0%)	34(14.5%)
Stomatitis/pharyngitis	18(7.7%)	3(1.3%)	1(0.4%)	0(0%)	22(9.4%)
Nausea	31(13.2%)	9(3.8%)	1(0.4%)	0(0%)	41(17.5%)
Vomiting	15(6.4%)	10(4.3%)	3(1.3%)	0(0%)	28(12.0%)
Abdominal pain or cramping	13(5.6%)	8(3.4%)	7(3.0%)	2(0.8%)	30(12.8%)
Bilirubin*	5(2.1%)	2(0.8%)	1(0.4%)	0(0%)	8(3.4%)
SGOT (AST)*	6(2.6%)	5(2.1%)	1(0.4%)	0(0%)	12(5.1%)
Alkaline phosphatase*	8(3.4%)	4(1.7%)	2(0.8%)	0(0%)	14(6.0%)
Diarrhea	45(19.2%)	10(4.3%)	3(1.3%)	0(0%)	58(24.8%)
Dry Skin	18(7.7%)	1(0.4%)	0(0%)	0(0%)	19(8.1%)
Hypertension	6(2.6%)	3(1.3%)	5(2.1%)	0(0%)	14(6.0%)

Includes all hematologic and hepatic toxicities and other non-hematologic toxicities with an incidence of ≥ 5%.
*These lab findings were reported as adverse event by the investigator and not by lab measurements.
All events are included in both the run-in and the randomized phase.

There were no fatal drug-related AE reported, the incidence of life-threatening (grade 4) events was 0.8% and severe toxicities was 23%, over half of which were dermatologic or nausea/vomiting/constitutional. In summary, at 400 mg bid continuously BAY 43-9006 is extremely safe with little severe toxicity, which is generally manageable by dose reduction if it occurs.

1.5 Recommended Phase II Dose

Available Phase 1 safety data indicate that 400 mg bid is the maximally tolerated dose. Increasing the dose from 400 mg bid to 600 mg bid caused a significant increase in Grade 3 and 4 toxicities, including significant skin toxicities. Consistent with this, there is a significant increase in the number of patients requiring dose-reduction or withdrawal of study drug due to adverse events/toxicity at the 600 mg bid dose level compared to the 400 mg bid dose-level. Evaluation of safety data across various schedules did not show significant differences in toxicities between the continuous and intermittent schedules. The continuous uninterrupted schedule was chosen over intermittent schedules due to preclinical data indicating possible

advantages following continuous dosing. Safety data indicated the use of 400 mg bid dose-level administered using the uninterrupted schedule for Phase 2/3 trials.

Pharmacokinetic data showed an increase in exposure with increasing dose from 100 mg bid up to the 600 mg bid dose-level. With a doubling of dose from 100 mg bid to 200 mg bid, there was a 55% increase in exposure. There was a 40% increase in exposure with a doubling of the dose from 200 mg bid to 400 mg bid. As the dose is increased, the average exposure increases and the fraction of patients below the average efficacious exposure decreases. Using the highest tolerated dose of 400 mg bid allows the average exposure to be higher than the average exposure at the lower dose levels of 200 mg bid and 100 mg bid. Therefore, a larger fraction of patients would in the range of efficacious exposures. Pharmacokinetic data support the use of 400 mg bid over doses of 200 or 100 mg bid.

Available Phase 1 antitumor activity data indicate similar activity at the 400 mg bid (1 partial response and 1 minor response) and the 600 mg bid (1 partial response and 1 minor response) dose-levels. There were no responders at the 100 mg bid and the 200 mg bid dose-level. Antitumor activity data did not indicate a preference for choosing 600 mg bid over 400 mg bid. Further evidence of efficacy in the Phase II randomized discontinuation trial, hepatoma, and RCC trials support 400 mg bid as the recommended single agent dose.

2.0 Correlative Studies of Elevated IFP

2.1 Rationale

A major challenge of targeted therapies clinically is the optimization of dose and schedule for combination therapy in individual patients. Although serial tumor biopsies can provide the necessary information, these are difficult to obtain. Therefore, there is an urgent need for imaging technologies and surrogate markers that permit specific phenotypic changes to be quantified during therapy. Efforts are underway to adapt magnetic resonance imaging, computed tomography (CT), positron emission tomography (PET), ultrasound and various optical techniques. We recently documented in patients with colorectal cancer that bevacizumab, a VEGF-blocking antibody, is a potent anti-angiogenic agent. Twelve days after one injection of bevacizumab, functional CT measurements demonstrated a significant reduction in tumor blood volume and flow, which was associated with a significant decrease in tumor vascular density.[14] Bevacizumab also increased the fraction of the tumor vessels covered by pericyte-like cells and decreased the tumor IFP, which are indicators of a « normalized » vasculature and improved therapeutic agent delivery. In pre-clinical studies in mice, inhibition of VEGF receptor-2 signaling with a blocking antibody improved the penetration of large molecules in tumors, which was most likely caused by the reduction in tumor IFP and increased hydrostatic pressure gradient from the vascular to the interstitial space.[15]

2.2. Angiogenesis and Biological/Physiologic Markers

2.2.1 Perfusion CT scans have been employed in assessing responses of soft tissue sarcomas and/or other malignancies to radiation therapy and/or chemotherapy. In recent years, all the cross sectional modalities, including CT have shown potential as functional imaging tools. Functional CT has been successfully applied to a number of organs such as the brain, heart, liver and kidneys. Applications in tumor imaging have included studies showing it may improve the detection of hepatic metastases because of abnormal blood flow changes and measure capillary permeability in lymphoma nodes. The development of fast spiral scanners

with excellent spatial resolution makes CT highly suited to the investigation of functional indexes. The combination of high anatomic resolution with functional information enables parametric images to be constructed, an approach particularly suitable for imaging the heterogeneous blood supply of many malignancies.

2.2.2 Targeting angiogenic vessels requires adequate methods for the assessment of the biologic effect of various new drugs developed to control cancer progression. Tumor angiogenesis is evaluated mainly by measuring microvessel density (MVD) in biopsy specimens using immunohistochemistry. Predicting and/or assessing accurately the efficacy of antiangiogenic therapies by this method is hampered by the heterogeneity of tumors, and by the difficulty to obtain specimens at multiple time points during treatment. The correlation of MVD with the clinical outcome is still uncertain in most tumor types.[16] In patients with soft tissue sarcoma no relationship was found between clinical outcome and MVD.[17] Interestingly, in the same study high tumor levels of VEGF were correlated with enhanced probability of recurrence and metastasis, and reduced overall survival.

2.2.3 The number of circulating endothelial cells (CECs), measured by flow cytometry, is significantly increased in the peripheral blood of untreated lymphoma and breast cancer patients.[18] Furthermore, in lymphoma patients achieving complete remission after chemotherapy, the number of CECs was reduced to the values observed in healthy controls, and activated CECs were found to decrease in breast cancer patients evaluated before and after quadrantectomy.[19]

2.2.4 The strong correlation observed between the number of CECs and tumor volume indicates that CECs increase with tumor progression.[17] The evaluation of CEC kinetics and viability before and after drug therapy demonstrated that in control animals, most CECs seem to have initiated an apoptotic program, whereas CEC viability is markedly improved in tumor-bearing mice. Moreover, a correlation was found between CEC and the levels of human VEGF (produced by tumor cells) in plasma, suggesting that the anti-apoptotic properties of VEGF may also play a role in determining the number of CECs in tumor-bearing mice.[17] The increase in CECs in cancer patients has been attributed to shedding from tumor vessels[20] or from distant uninvolved vessels that are activated by cytokines produced by the tumor, such as bone marrow.[21]

2.2.5 In conclusion, CEC evaluation is a relevant surrogate, non-invasive angiogenesis marker that may contribute to the existing panel of angiogenesis assays. Importantly, the measurement of CEC viability seems a useful, noninvasive tool to evaluate the efficacy of targeted antiangiogenic drugs in clinical trials. Finally, considering that the number of CECs correlate with tumor volume and VEGF levels in plasma, it might be particularly useful as a relevant prognostic factor in a clinical trial involving VEGF blockade. In a phase I clinical trial we found that Bevacizumab reduced the number of viable CECs and progenitor cells in patients with rectal cancer.[12]

2.2.6. Studies from our group and other investigators have shown that the interstitial fluid pressure (IFP) in colon, breast, lung, head and neck, cervix and skin tumors is significantly higher than in normal tissues.[22,23,24,25,26,27] To determine if intratumoral pressure could be used as a marker of tumor response, we measured pressure before and during radiation treatment in patients with cervical cancer. In general, a decrease in tumor pressure was associated with a better response to radiation. In women with a poor tumor response there was an increase or no change in pressure.[20] A recent study that included more than 100 patients has shown that radiation therapy improves the survival of patients with tumor pressures below 20 mm Hg.[25]

These findings demonstrate that intratumoral pressure could predict treatment outcome and that pressure could be used to determine if patients should be treated more aggressively. Response to anti-angiogenic therapy could also be predicted by modifications in interstitial fluid pressure. VEGF and VEGF receptor-2 blocking antibodies significantly decrease the IFP of human tumors implanted in mice and in rectal tumors in patients.[28,12]

2.3 Summary for study of sorafenib/BAY 43-9006 and Correlative Biology

Sorafenib was selected for development based on the inhibition of the *raf* kinase enzyme and is a particularly exciting agent for targeting elevated tumor IFP. The *ras/raf* signaling pathway is an important mediator of responses to growth signals and angiogenic factors. This pathway is often aberrantly activated in human tumors due to presence of activated *ras*, mutant *b-raf*, or overexpression of growth factor receptors. Sorafenib is a potent inhibitor of *c-raf* and wild-type and mutant *b-raf* ^{V599E} *in vitro* and several RTKs that are involved in tumor angiogenesis (humanVEGFR-2, murine (m)VEGFR-2, mVEGFR-3, mPDGFR-β) at nanomolar concentrations. In cellular assays, sorafenib was found to be a potent inhibitor of human and mVEGFR-2, mVEGFR-3, and mPDGFR-β. receptor phosphorylation. In human tumor xenograft samples stained for CD31 (PECAM) expression, there was a dramatic reduction of tumor neo-vascularization. These data suggest that sorafenib may inhibit tumor progression through multiple mechanisms, directly inhibiting tumor cell proliferation dependent on activation of the MAPK pathway and indirectly by inhibiting tumor angiogenesis or neo-vascularization through inhibition of VEGFR-2, VEGFR3, and/or PDGFR-β. Activation of *raf-1* in EC as a downstream event of both VEGFR-2 and bFGF stimulation protects against apoptosis and is a pivotal regulator of EC survival during angiogenesis[30]. Sorafenib acts at several critical points in angiogenesis and vascular permeability, both at the EC surface receptor and the downstream signal cascade level and may be uniquely effective in cancer therapy.

The purpose of this study is to determine if the combined VEGF-R2, PDGFRβ, *raf-1/B-raf* inhibitor sorafenib can decrease IFP in soft tissue sarcomas. Measurement of IFP in the clinic will be correlated with other properties of PDGFβ and VEGF on EC and tumor vasculature. To provide a comprehensive investigation of the inhibitory effect of sorafenib on EC RTKs, a series of interrelated and complementary correlative studies will be performed. Microscopic analysis of microvascular density, pericyte coverage, and endothelial cell apoptosis, tumor blood flow and permeability, tumor glucose utilization, and circulating endothelial cells (CECs) will be determined. The expression of EC specific mRNA pre- and post- sorafenib will be performed on tumor EC. Pharmacokinetics will be performed in all patients to determine if the variable systemic exposure seen with sorafenib correlates with these biologic markers. This will be the most thoroughly detailed study to date of the biological effects of sorafenib in humans.

3.0 Objectives

3.1 Primary Objectives

1. To determine if the combined VEGF-R2/PDGFRβ inhibitor BAY 43-9006/ sorafenib can decrease interstitial fluid pressure (IFP) in soft tissue sarcomas.

2. To investigate the effects of BAY 43-9006/sorafenib on tumor blood flow, circulating endothelial cells, vascular density and pericyte coverage.

3. To characterize the pharmacokinetics of BAY 43-9006/sorafenib in sarcoma patients.

3.2 Secondary Objectives

1. To describe any preliminary evidence of anti-tumor activity.

2. Assess whether there are any significant relationships between systemic drug exposure and drug-related toxicity or biological effect.

4.0. Patient Selection

4.1 Eligibility Criteria

1. As of 5/30/07, no subjects will accrue to Treatment Group I.
   There are two groups of patients eligible for this study. Treatment group 1 consists of patients with extremity sarcomas other than potentially curable osteosarcoma or Ewing's sarcoma who are candidates for potentially curative surgery. Treatment group 2 consists of patients with metastatic or inoperable sarcoma, for which there is no known curative or survival prolonging palliative therapy, or failure of these therapies. Patients must have at least one site of measurable disease by radiologic imaging techniques. Patients must have at least one palpable tumor mass with no overlying viscera which is amenable to biopsy. The tumor mass should be approximately 2 cm or greater in diameter. Patients with smaller palpable tumors are eligible if participation is approved by the treating surgeon after discussion with the study chairperson.

2. Age ≥ 18 years.

3. Life expectancy ≥ 2 months.

4. ECOG performance status ≤ 2.

5. Pretreatment laboratory data, obtained within 14 days of study entry, must meet the following criteria:

ANC	≥1,500/mm^3
Platelets	≥100,000/mm^3
SGOT	≤2.5-times the upper limit of normal (ULN)
SGPT	≤2.5-timesULN
Total Bilirubin	≤ULN
Serum creatinine	≤ 1.5-times ULN

6. ≥ 3 weeks since major surgery unrelated to study disease (sarcoma).

7. ≥ 3 weeks since chemotherapy or radiation therapy (6 weeks for nitrosourea or mitomycin C chemotherapy).

8. No prior treatment with sorafenib (BAY 43-9006) or specific inhibitors of MAPK.

kinase pathways are permitted. A previously irradiated tumor site cannot be used for clinical or correlative measurements, although irradiation to sites other than a measurable site is permitted. There are no limitations on the extent or type of prior therapy received by the patient other than the time intervals indicated in the above and demonstrating complete recovery from any adverse effects associated by satisfying all relevant eligibility criteria.

9. Patients who are on warfarin anticoagulation are allowed to participate as long as they are converted to a low molecular weight heparin (e.g. lovenox) from study entry until at least day 56.

10. Women of childbearing potential must not be pregnant or lactating. All women of childbearing potential (age < 50, LMP < 12 months ago) must have a negative serum or urine pregnancy test (minimum sensitivity 25 IU/L of β-HCG) within 72 hr prior to receiving the study medication. BAY43-9006 has antiproliferative effects, which may be harmful to the developing fetus or nursing infant.

11. Fertile males and females must use adequate contraception.

12. Signed informed consent.

4.2 Exclusion Criteria

Patients will be considered ineligible for participation in the study for the following reasons:

1. Ewing's sarcoma or osteosarcoma that is potentially curable with surgery, chemotherapy, and/or radiation therapy.

2. Active brain metastases including evidence of cerebral edema by CT scan or MRI, or progression from prior imaging study, any requirements for steroids, or enzyme–inducing anti-convulsant agents, or clinical symptoms of/from brain metastases. Patients with treated and/or stable brain metastasis who are asymptomatic can be enrolled, if otherwise eligible.

3. Any uncontrolled serious medical or psychiatric illness. Particular note is given to uncontrolled hypertension (discretion left to investigators) and significant proteinuria > 1 gm/24 hr (does not require quantitation in absence of clinical indication).

4. Patients receiving other investigational agents.

5. HIV patients receiving combination anti-retroviral therapy are excluded because of potential pharmacokinetic interactions.

5.0 Registration

The Dana-Farber Cancer Institute (DFCI) will serve as the coordinating center for this study. A patient may be entered in the study once it has been determined that the patient meets all eligibility criteria and informed consent has been obtained. Final approval for registration and dose assignment will be made by the overall study chairperson, Dr. Jeffrey A. Morgan, MD (phone: 617-632-5204; pager: 632-2337, # 41215). Eligible patients must be registered through the DFCI Quality Assurance Office for Clinical Trials (QACT) by phone (617-632-3761) or fax (617-632-2295). Signed institutional consent forms, eligibility checklists, and on-study laboratory results must be forwarded to the QACT at the time of registration.

6.0 Drug Treatment plan

6.1 Pharmaceutical information

BAY 43-9006 tosylate (NSC 724772, IND 69,896)

Chemical Name 4- {4-[3-(4-chloro)-3-triflouromethyl-phenyl) ureido]phenoxy} -pyridine-2 carboxylic acid methylamide-4-methylbenzensulfonate.

Figure 6.1.1: – Structure of BAY 43-9006 tosylate (BAY 54-9085)

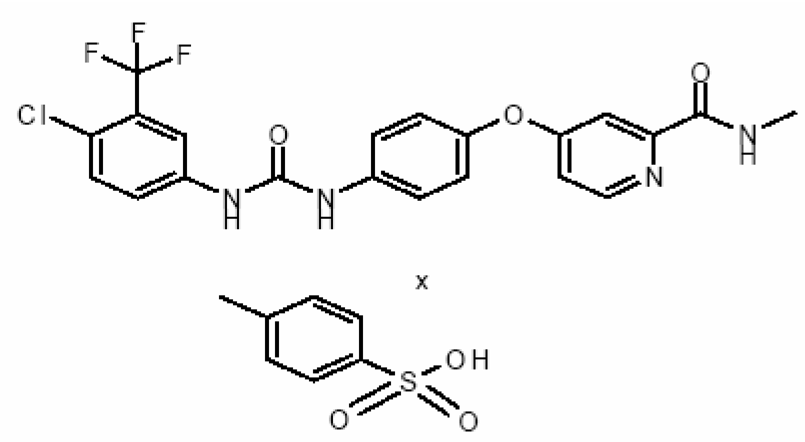

Other Names: BAY 54-9085 is the tosylate salt of BAY 43-9006; sorafenib

Classification: Kinase inhibitor (Raf, VEGF-R, and PDGF-R)
Mechanism of Action: The ras/raf signaling pathway is an important mediator of responses to growth signals and angiogenic factors. This pathway is often aberrantly activated in human tumors due to presence of activated ras, mutant b-raf, or over expression of growth factor receptors.

BAY 43-9006 is a potent inhibitor of c-raf, and wild-type and mutant b-raf in vitro. Additionally, further characterization of BAY 43-9006 tosylate revealed that this agent inhibits several

receptor tyrosine kinases (RTKs) that are involved in tumor progression (VEGF-R, PDGF-R, Flt, and c-KIT) and p39α, a member of the MAPK family.

Molecular formula: $C_{12}H_{16}ClN_4O_3$ X $C_7H_8O_3S$

M.W.: BAY 43-9006 tosylate: 637 Daltons; BAY 43-9006 (free base): 465 Daltons

Approximate Solubility: 0.19mg/l00mL in 0.1 N HCl, 453mg/100mL in Ethanol, and 2971 mg/mL in PEG 400.

6.1.1 How Supplied: BAY 43-9006 tosylate is a supplied as an immediate-release film-coated, round, and salmon color tablet containing 200 mg of the free base, BAY 43-9006, and the excipients croscarmellose sodium, microcrystalline cellulose, hydroxypropylmethylcellulose, sodium lauryl sulfate, and magnesium stearate. The film-coat consists of hydroxypropylmethylcellulose, polyethylene glycol, titanium dioxide and red iron oxide. The film coating has no effect on the rate of release of the active BAY 43-9006 tosylate.

BAY 43-9006 tosylate 200 mg tablets are supplied in bottles of 140 tablets.

Storage: Store at controlled room temperature (15°C – 25 °C). Storage conditions should not exceed 25°C.

Stability: Stability studies with the 200 mg dose form are ongoing. The current shelf life is 24 months when stored at controlled room temperature.

Route(s) of Administration: Orally

6.1.2 Reported Adverse Events and Potential Risks:

Refer to Section 6.11 for BAY43-9006 adverse event information.

6.1.3 Method of Administration: BAY 43-9006 tosylate should be taken with at least 250 mL of water. Study drug may be taken either with a moderate fat meal (approximately 30% of calories from fat) or without food. After a dose, patients do not have to wait before eating. Subjects should not make up missed or vomited doses.

6.1.4 Potential Drug Interactions: BAY-9006 tosylate is metabolized by the P450 CYP3A enzyme and has been shown in preclinical studies to inhibit multiple CYP isoforms. Therefore, it is possible that BAY-9006 tosylate may interact with drugs that are metabolized by the P450 CYP isoenzymes or with drugs that inhibit CYP 3A. Close monitoring is recommended for patients taking agents with narrow therapeutic indices and metabolized by the liver, such as warfarin, phenytoin, quinidine, carbamazepine, Phenobarbital, cyclosporine, and digoxin. Additionally, BAY-9006 tosylate is 97% to 99% protein-bound; however, no drug interactions have been reported in studies, thus far.

6.1.5 BAY 43-9006 Availability, Ordering and Accountability:

BAY43-9006 is provided to the NCI under a Clinical Trials Agreement (TA) between Bayer Corp. and Onyx Pharmaceuticals and the DCTD, NCI.

Agent Ordering: NCI-supplied agents may be requested by the Principal Investigator (or their authorized designees) at each participating institution. Pharmaceutical Management Branch (PMB) policy requires that the agent be shipped directly to the institution where the patient is to be treated. PMB does not permit the transfer of agents between institutions (unless prior approval from the PMB is obtained). Completed Clinical Drug Requests (NIH-986) should be submitted to the PMB by fax (301) 480-4612 or mailed to the Pharmaceutical Management Branch, CTEP, DTCD, NCI, 9000 Rockville Pike, EPN Rm. 7149 Bethesda, MD 20892.

Agent Accountability: The Investigator, or a responsible party designated by the Investigator, must maintain a careful record of the inventory and disposition of all agents received from DCTD using the NCI Drug Accountability Record Form.

6.2 Pretreatment Evaluations

1. Physical examination including vital signs (including a neurological exam, weight, blood pressure, temperature, pulse, and respiratory rate) and documentation of ECOG performance status within 14 days of registration.

2. Laboratory data including CBC with differential, platelets, electrolytes, albumin, Ca^{2+}, Mg^{2+}, BUN, creatinine, total bilirubin, SGOT, SGPT, alkaline phosphatase, LDH and urinalysis within 14 days of registration.
3. Appropriate imaging studies necessary for tumor measurements (*eg* plain radiograph, CT scan, MRI, nuclear medicine scan) within 28 days of registration.

4. ECG showing no clinically significant (unstable or uncontrolled) atrial or ventricular arrhythmias, including 2°/Mobitz type 2 or 3° heart block.

6.3 On-Study Evaluations

1. A physical examination including vital signs (including a neurological exam, weight, blood pressure, temperature, pulse, and respiratory rate), performance status, CBC with differential, platelets, electrolytes, albumin, BUN, creatinine, Ca^{2+}, Mg^{2+}, SGOT, SGPT, alkaline phosphatase, total bilirubin, LDH and urinalysis will be performed weekly during cycle 1, as well as prior to each additional cycle of therapy. These tests will also be performed every other week (cycles 2 through 4), monthly (cycles 5 and beyond), and one month after the last dose of BAY 43-9006 has been administered.

2. Patients with measurable disease will be rescanned to measure disease after even numbered cycles of therapy or until documentation of tumor progression.

3. An assessment of adverse events and toxicities will be performed weekly during therapy, prior to each additional cycle of therapy, every two weeks (cycles 2 through 4) while on therapy, monthly (cycles 5 and beyond), and one month after the last dose of BAY 43-9006 has been administered. Toxicities will be categorized and graded according to the NCI CTCAE Version 3.0 (see section 6.11) and recorded.

4. This study will be monitored by the Clinical data Update System (CDUS), version 3.0. Cumulative CDUS data will be submitted quarterly to CTEP by electronic means. Reports are due January 31, April 30, July 31 and October 31.

5. Blood specimens for pharmacokinetic studies and evaluating surrogate markers of pharmacological activity will be obtained during the first cycle of therapy as described in section 12.0.

6.4 Drug Administration and Treatment Plan

1. Therapy will be administered on an outpatient basis. Patients will keep a drug diary to be returned to the study staff every cycle.

2. Each dose of BAY 43-9006 will be two (2) 200-mg tablets taken orally two times a day (approximately 12 hours apart) with at least 250 mL of water.. Study drug may be taken either with a moderate fat meal (approximately 30% of calories from fat) or without food. After a dose, patients do not have to wait before eating. Subjects should not make up missed or vomited doses.

3. The dose of BAY 43-9006 to be administered two times a day daily will be 400 mg. This is the recommended Phase II dose (RP2D) of BAY 43-9006 when given as a single agent. The length of each cycle is 14 days (Treatment Group I) and 28 days (Treatment Group II).

4. In calculating body surface areas, actual heights and weights should be used. These calculations will be used for pharmacokinetic analysis and not dosing.

5. **Treatment Group I:** Patients with surgically resectable bone or soft tissue sarcomas of the extremity (except localized osteosarcoma and Ewing's sarcoma) will have tumor pressures recorded, an incisional or core biopsy for confirmation of diagnosis and correlative studies, CBC, blood chemistries, ECG, and digital contrast-enhanced magnetic resonance imaging (dce-MRI) or functional CT scan at the discretion of the treating surgeon. Patients will then receive BAY 43-9006 400 mg two times a day for two weeks. Patients will have limited pharmacokinetic (PK) sampling on Day 14 (see Section 12.0). Note that patients will skip the second (evening) dose of BAY43-9006 on the day of PK sampling. Repeat dce-MRI or CT, ECG, CBC, and blood chemistries will be performed prior to surgical resection (on or about day 15 as scheduling permits). Patients should stop taking BAY43-9006 at least 24 hours before surgery. In the Operating Room, prior to surgical resection, repeat tumor pressures

will be recorded. 200 mg of the tumor specimen will be reserved for magnetic resonance quantitation of BAY 43-9006. The remainder of the specimen will reserved for clinical and research pathologic analysis. Patients who demonstrate a clinically and pathologically significant response ($\geq$ 25% reduction in tumor size or $\geq$ 25% necrosis in the surgical specimen) may continue to receive BAY 43-9006 at the investigators discretion, subject to repeat assessment for progressive disease as Group II. This re-treatment may begin no earlier than 15 days after surgery but may be delayed until resolution of all post-operative complications (Section 6.5.3).

6. **Treatment Group II**: Patients with sarcomas for which there is no chemotherapy of established benefit, with tumor accessible for repeat measurements of IFP and tissue biopsy and who will consent to serial biopsies/measurements will also be eligible to enroll. All tumors need to be palpable with no overlying viscera. Patients will have tumor pressures recorded, have an incisional or core biopsy for confirmation of diagnosis and correlative studies, and CBC, blood chemistries, ECG, and functional CT scan imaging of blood flow. Patients in treatment group II will receive BAY 43-9006 400 mg two times a day for 8 weeks. Patients will have pharmacokinetic (PK) sampling on Days 28 and 56 (see Section 12.0). Note that patients will skip the second (evening) dose of BAY43-9006 on these days of extensive PKs. On day 28 (+/- 3 days) and day 56 (+/- 3 days), patients will have repeated functional CT, IFP measurements, and repeat biopsies (day 28 (+/- 3 days) only), with handling as in treatment group I. There will be an additional MD appointment within one week of Day 28 to review the results of the Day 28 assessments. If any of these patients demonstrate clinical benefit (response or stable disease), these patients will be eligible to continue BAY 43-9006/sorafenib subject to repeat response assessment. This group may also include patients deemed clinically appropriate for non-curative palliative surgery.

7. Treatment group II will seek to accrue at least 6 patients fully evaluable in each of the pharmacodynamic/correlative studies and pharmacokinetic assays.

8. Patients experiencing a subjectively intolerable toxicity for whom continued therapy is desirable or appropriate may interrupt therapy for up 14 days or until resolution of all objective and subjective toxicities/symptoms to $\leq$ grade I or to baseline, at the investigator’s discretion. Patients will resume therapy at 200 mg two times a day. A second reduction to 200 mg po once daily is permitted for the above reasons, subject to the same conditions.

9. With initiation of study therapy or an increase in dose the dose of sorafenib, monitor BP at least weekly until stable on study therapy or at least for the first 4 weeks.

6.5 Patient Re-treatment

1. It is intended that patients receive a minimum of 14 days (Group I) or 56 days (Group II), in the absence of unacceptable toxicity. Patients with stable disease or objectively responding disease will be a candidate for additional cycles of sorafenib at the discretion of the Investigator. Patients with progressive disease will be taken off study. Sorafenib may be continued for a maximum of 6 months for Treatment Group 1 where it is pseudo-adjuvant. Treatment Group 2 may be treated with an unlimited number of sorafenib cycles.
2. Patients must continue to meet eligibility requirements with respect to performance status, hematologic parameters, hepatic and renal function criteria before receiving a second or subsequent cycle. Additionally, all toxicities that are considered as possibly being related to BAY 43-9006 must have recovered to baseline or ≤ grade 1 prior to weekly re-treatment or beginning a second or subsequent cycle of therapy.

3. The administration of additional cycles of therapy will be delayed if a patient fails to fully meet the criteria for re-treatment by day 28 of the current cycle, in which case the patient will be re-evaluated after one week. Subsequent cycles of therapy may be delayed for two weeks to allow for full recovery from toxicity. Any patient not able to begin the next cycle of therapy within two weeks of the planned treatment date may be allowed additional time to resolve toxicity at the discretion of the investigator. This is meant to apply primarily to patients in Group I who may have indications for continued treatment with BAY 43-9006 after surgery but who may require additional time to fully recover from surgery (section 6.7) or adjuvant radiation therapy. In exceptional circumstances, this may apply to Group II patients if the investigator and principal investigator agree.
4. The dose to be used for additional cycles for both treatment groups is the current dose for the subject.

6.6 Dose Modifications

1. Patients experiencing a unacceptable toxicity during any treatment cycle may be retreated with BAY 43-9006 at a dose reduced to 200 mg two times a day, provided that all toxicities considered related to BAY 43-9006 have recovered to baseline or ≤ grade 1.
   A second dose reduction to 200 mg po once daily is permitted for the same reasons. Any patient who requires a dose reduction will be treated with a reduced dose for any subsequent courses of BAY 43-9006; that is, there will be no dose re-escalation.

6.7 Duration of Treatment

Patients who demonstrate objective tumor response or stable disease will continue to receive treatment as long as clinical benefit is observed (Treatment Group II).

Patients in Treatment Group I who demonstrate a clinically and pathologically significant response ($\geq$ 25% reduction in tumor size or $\geq$ 25% necrosis in the surgical specimen) may continue to receive BAY 43-9006 at the investigators discretion, subject to repeat assessment for progressive disease as in Group II. BAY 43-9006 (sorafenib) may be continued for a maximum of 6 months.

6.8 Withdrawal from Therapy

Patients will be withdrawn from protocol therapy if any of the following occurs:

1. Any toxicity that fails to resolve to $\leq$ grade 1 within time allowed (approximately 14 days or longer at the Investigator's discretion). Additionally, subjects must be taken off study therapy after a GI-perforation.

2. Progressive disease requiring alternate therapy.

3. The patient or their primary physician requests that the patient be withdrawn from therapy.

4. Patient non-adherence with the protocol. This includes failure to administer 3 or more doses of BAY43-9006 (sorafenib), except in cases of adverse event. This also includes (but is not limited to) repeated missing of appointments or assessments except due to adverse events or circumstances deemed beyond the patient's control or any behavior which, in the opinion of the Investigators, threatens the safety of the patient or other patients, or compromises the integrity of the study.

6.9 Concurrent Supportive Care

1. Narcotics and antiemetics may be given as needed.

2. The use of herbal supplements other than St. John's Wort will be permitted at the discretion of the treating physician.

3. The administration of radiation is permitted only as an adjuvant in patients receiving surgery for an extremity sarcoma. Patients requiring radiotherapy will not receive concurrent BAY 43-9006 but may resume the study agent at the resolution of all radiation related toxicity if otherwise indicated.

4. Hand-foot syndrome is one of the major toxicities of sorafenib/BAY43-9006 therapy. Patients who develop hand-foot syndrome may receive topical emollients (such as Aquaphor) as well as topical steroids or antihistamine agents if appropriate. Vitamin B6 (pyridoxine; 50 - 150 mg orally daily) may also be used.

5. Management of Treatment-emergent Hypertension:

Grade of Event (CTCAE v.3)	Management/ Next Dose
grade 1	Consider increased BP monitoring or begin anti-hypertensive therapy and continue agent
grade 2 asymptomatic and diastolic BP < 110 mm Hg	Begin anti-hypertensive therapy and continue agent
grade 2 symptomatic/ persistent<br>OR<br>diastolic BP ≥ 110 mm Hg<br>OR<br>grade 3	1. Agent should be held* until symptoms resolve and diastolic BP ≤ 100 mm Hg; also treat patient with anti-hypertensives and when agent is restarted, reduce by 1 dose level.**<br>2. If diastolic BP is not controlled (≤ 100) on therapy, reduce another dose level ***
grade 4	Discontinue protocol therapy

* Patients requiring a delay of > 2 weeks should go off protocol therapy.
** May be able to resume full dose later.
*** Patients requiring > 2 dose reductions should go off protocol therapy.

Current CTCAE definitions used by CTEP:

Grade 1: asymptomatic, transient (< 24 hours) increase by > 20 mmHg (diastolic) or to >150/100 if previously WNL; intervention not indicated
Grade 2: recurrent or persistent (> 24 hours) or symptomatic increase by > 20 mmHg (diastolic) or to > 150/100 if previously WNL; monotherapy may be indicated
Grade 3: requiring more than one drug or more intensive therapy than previously
Grade 4: life threatening (e.g. hypertensive crisis)

6.10 Toxicity

6.10.1 Evaluations

1. All toxicities will be evaluated and graded according to the NCI CTC Version 3.0 for toxicity and adverse event reporting. A copy of the CTCAE version 3.0 can be downloaded from the CTEP home page (http://ctep.info.nih.gov). All appropriate treatment areas should have access to a printed copy of the CTCAE Version 3.0.

2. Toxicities should be managed with appropriate medical care.

3. All subjects will be monitored for gastrointestinal toxicities, particularly as they may relate to the onset or development of gastrointestinal perforations. Subjects who develop a GI perforation must be taken off protocol therapy.

6.11 Adverse Event Reporting Requirements

Comprehensive Adverse Events and Potential Risks List (CAEPR) for Sorafenib (BAY 43-9006, NSC 724772)

The Comprehensive Adverse Event and Potential Risks list (CAEPR) provides a single list of reported and/or potential adverse events (AE) associated with an agent using a uniform presentation of events by body system. In addition to the comprehensive list, a subset, the Agent Specific Adverse Event List (ASAEL), appears in a separate column and is identified with ***bold*** and ***italicized*** text. This subset of AEs (ASAEL) contains events that are considered 'expected' for expedited reporting purposes only. Refer to the 'CTEP, NCI Guidelines: Adverse Event Reporting Requirements' http://ctep.cancer.gov/reporting/adeers.html for further clarification. *Frequency is provided based on 1376 patients*. Below is the CAEPR for sorafenib.

Version 2.1, June 23, 2006[1]

Adverse Events with Possible Relationship to Sorafenib (BAY 43-9006) (CTCAE v3.0 Term) [n=1376 patients]			'Agent Specific Adverse Event List' (ASAEL)
**Likely (>20%)**	**Less Likely (<=20%)**	**Rare but Serious (<3%)**	
ALLERGY/IMMUNOLOGY			
	Allergic reaction/hypersensitivity (including drug fever)		
BLOOD/BONE MARROW			
	Hemoglobin		
	Leukocytes (total WBC)		
	Lymphopenia		
	Neutrophils/granulocytes (ANC/AGC)		
	Platelets		
CARDIAC GENERAL			
	Hypertension		***Hypertension***
CONSTITUTIONAL SYMPTOMS			
Fatigue (asthenia, lethargy, malaise)			***Fatigue (asthenia, lethargy, malaise)***
	Fever (in the absence of neutropenia, where neutropenia is defined as ANC <1.0 x 10e9/L)		
	Rigors/chills		
	Weight loss		
DERMATOLOGY/SKIN			
	Dermatology/Skin - Other (non-life threatening squamous cell carcinoma of skin: keratoacanthoma type)		
	Dermatology/Skin - Other (other)		
	Dry skin		

	Flushing		
	Hair loss/alopecia (scalp or body)		
	Hypopigmentation		
	Nail changes		
	Pruritus/itching		
Rash/desquamation			***Rash/desquamation***
Rash: hand-foot skin reaction			***Rash: hand-foot skin reaction***
GASTROINTESTINAL			
	Anorexia		
	Ascites (non-malignant)		
	Constipation		
	Dehydration		
Diarrhea			***Diarrhea***
	Dysphagia (difficulty swallowing)		
	Flatulence		
	Heartburn/dyspepsia		
	Mucositis/stomatitis (functional/symptomatic): pharynx		
	Nausea		
		Perforation, GI - NOS	
	Vomiting		
HEMORRHAGE/BLEEDING			
	Hemorrhage GI - Select		
	Hemorrhage GU - Select		
HEPATOBILIARY/PANCREAS			
	Pancreatitis		
INFECTION			
	Febrile neutropenia (fever of unknown origin without clinically or microbiologically documented infection)(ANC <1.0 x 10e9/L, fever >=38.5 degrees C)		
	Infection with unknown ANC - Select		
METABOLIC/LABORATORY			
	Albumin, serum-low (hypoalbuminemia)		
	Alkaline phosphatase		
	ALT, SGPT (serum glutamic pyruvic transaminase)		
	Amylase		
	AST, SGOT (serum glutamic oxaloacetic transaminase)		
	Bilirubin (hyperbilirubinemia)		
	GGT (gamma-glutamyl transpeptidase)		
	Glucose, serum-high (hyperglycemia)		

	Lipase		
	Metabolic/Laboratory - Other (blood elastase)		
	Phosphate, serum-low (hypophosphatemia)		
NEUROLOGY			
	Neuropathy: sensory		
PAIN			
	Pain - abdomen NOS		
	Pain - joint		
	Pain - muscle		
	Pain NOS		
PULMONARY/UPPER RESPIRATORY			
	Hypoxia		
	Pleural effusion (non-malignant)		
	Pneumonitis/pulmonary infiltrates		
	Pneumothorax		
RENAL/GENITOURINARY			
	Renal failure		
SYNDROMES			
	Flu-like syndrome		

[1]This table will be updated as the toxicity profile of the agent is revised. Updates will be distributed to all Principal Investigators at the time of revision. The current version can be obtained by contacting ADEERSMD@tech-res.com. Your name, the name of the investigator, the protocol and the agent should be included in the e-mail.

Also reported on BAY 43-9006 trials but with the relationship to BAY 43-9006 still undetermined:
ALLERGY/IMMUNOLOGY - linear IgA disease
CARDIAC ARRHYTHMIA - atrial flutter; supraventricular arrhythmia
CARDIAC GENERAL - cardiac ischemia/infarction; left ventricular systolic dysfunction
COAGULATION - INR; PTT; thrombotic microangiopathy
DERMATOLOGY/SKIN - erythema multiforme
GASTROINTESTINAL - ileus
HEMORRHAGE/BLEEDING - CNS hemorrhage; petechiae; pleural hemorrhage; splenic infarction
LYMPHATICS - limb edema
METABOLIC/LABORATORY - creatinine; hyperuricemia; hyponatremia
MUSCULOSKELETAL/SOFT TISSUE - arthritis
NEUROLOGY - anxiety; CNS ischemia; dizziness; encephalopathy; memory impairment; psychosis; syncope
OCULAR/VISUAL - diplopia; uveitis
PAIN - back pain; bone pain; chest/thorax pain; headache; limb pain
PULMONARY/UPPER RESPIRATORY - ARDS; dyspnea; voice changes
SEXUAL/REPRODUCTIVE FUNCTION - erectile dysfunction
VASCULAR - thrombosis/thrombus/embolism

Note: BAY 43-9006 in combination with other agents could cause an exacerbation of any adverse event currently known to be caused by the other agent, or the combination may result in events never previously associated with either agent.

Adverse events (AE) will use the descriptions and grading scales found in the revised NCI Common Terminology Criteria (CTCAE). This study will utilize the CTCAE version 3.0 for

adverse event reporting. All appropriate treatment areas will have access to a copy of the CTCAE version 3.0. The following adverse events are considered “Expected” and do not require expedited reporting through AdEERS: hypertension; fatigue (asthenia, lethargy, malaise); rash/desquamation; rash: hand-foot skin reaction; diarrhea.

Phase 2 and 3 Trials Utilizing an Agent under a CTEP IND: AdEERS Reporting Requirements for Adverse Events That Occur Within 30 Days[1] of the Last Dose of the Investigational Agent

	Grade 1	Grade 2	Grade 2	Grade 3		Grade 3		Grades 4 & 5[2]	Grades 4 & 5[2]
	Unexpected and Expected	Unex-pected	Expected	Unexpected		Expected		Unex-pected	Expected
				with Hospitali-zation	without Hospitali-zation	with Hospitali-zation	without Hospitali-zation		
**Unrelated Unlikely**	Not Required	Not Required	Not Required	10 Calendar Days	Not Required	10 Calendar Days	Not Required	10 Calendar Days	10 Calendar Days
**Possible Probable Definite**	Not Required	10 Calendar Days	Not Required	10 Calendar Days	10 Calendar Days	10 Calendar Days	Not Required	24-Hour; 5 Calendar Days	10 Calendar Days

1 **Adverse events with attribution of possible, probable, or definite that occur <u>greater</u> than 30 days after the last dose of treatment with an agent under a CTEP IND require reporting as follows:**

AdEERS 24-hour notification followed by complete report within 5 calendar days for:
- Grade 4 and Grade 5 unexpected events

AdEERS 10 calendar day report:
- Grade 3 unexpected events with hospitalization or prolongation of hospitalization
- Grade 5 expected events

2 Although an AdEERS 24-hour notification is not required for death clearly related to progressive disease, a full report is required as outlined in the table.

December 15, 2004

Note: All deaths on study require both routine and expedited reporting regardless of causality. Attribution to treatment or other cause must be provided.

- Expedited AE reporting timelines defined:
  - "24 hours; 5 calendar days" – The investigator must initially report the AE via AdEERS within <u>24 hours</u> of learning of the event followed by a complete AdEERS report within <u>5 calendar days</u> of the initial 24-hour report.
  - "10 calendar days" - A complete AdEERS report on the AE must be submitted within <u>10 calendar days</u> of the investigator learning of the event.

- Any medical event equivalent to CTCAE grade 3, 4, or 5 that precipitates hospitalization (or prolongation of existing hospitalization) must be reported regardless of attribution and designation as expected or unexpected with the exception of any events identified as protocol-specific expedited adverse event reporting exclusions.

- Any event that results in persistent or significant disabilities/incapacities, congenital anomalies, or birth defects must be reported via AdEERS if the event occurs following treatment with an agent under a CTEP IND.

- Use the NCI protocol number and the protocol-specific patient ID provided during trial registration on all reports.

7.0 Correlative Studies

7.1 Schedule of Correlative Studies Before, During and After BAY 43-9006 initiation

We will determine if BAY 43-9006/soranefib treatment of patients with soft tissue sarcomas will decrease the tumor IFP, and modify the vascular biology and proliferation and apoptotic profile of tumor cells. All correlative samples will be delivered to the Steele Labs at MGH, Yves Boucher, Ph.D., 617-726-4082.

Treatment Group I:

	**Pre-Tx**	**Day 15+ 7 days(surgery)**
**Biopsy**	**X**	**X**
**Plasma sample**	**X**	**X**
**Urine sample**	**X**	**X**
**Tumor IFP**	**X**	**X**
**Tumor imaging (functional CT or dce-MRI**	**X**	**X**

Treatment Group II:

	**Pre-Tx**	**Day 28 (+/- 3 days)**	**Day 56 (+/- 3 days)**
**Biopsy**	**X**	**X**	**None**
**Plasma sample**	**X**	**X**	**X**
**Urine sample**	**X**	**X**	**X**
**Tumor IFP**	**X**	**X**	**X**
**Tumor imaging (functional CT or dce-MRI)**	**X**	**X**	**X**

Sarcoma biopsy: Immunostaining for cell proliferation (PCNA), apoptosis (TUNEL), microvessel density (CD31) and pericyte coverage (αSMA).
Plasma samples (2 purple tops + 1 red top): Growth factors (VEGF, PDGFA, PDGFB) will be assayed. CECs will be extracted from whole blood prior to plasma separation and quantified for kinetics and viability of endothelial and progenitor cells.
Urine Sample: VEGF levels will be measured.
Tumor IFP: IFP will be measured with the wick-in-needle technique.
Radiologic Imaging: Tumor blood volume and flow, and vascular permeability surface area product will be measured with a functional CT scan approach or dce-MRI.

7.2 Methods

7.2.1 Functional imaging per MGH perfusion CT imaging guidelines.

7.3. Immunohistochemistry. Tumor vessel density, pericyte coverage of vessels, tumor cell proliferation and apoptosis will be measured in tumor biopsies obtained before and after the initiation of BAY 43-9006 treatment. The tumor samples will be fixed in paraformaldehyde and embedded in paraffin. To measure vessel density tumor sections will be stained with an antibody against human PECAM (Dako, Carpentria, CA). Pericyte coverage of the vessels will be

determined by double immunostaining of the biopsies for PECAM and αSMA (Sigma) as previously described (Willett et al., 2004). PCNA immunostaining will be used to identify proliferating cells, and TUNEL staining will be carried out following either published protocol or the manufacturer's recommendations. The state of EC VEGFR-2 phosphorylation will be measured by immunohistochemistry at baseline and after sorafenib/BAY43-9006 treatment, with the antibody supplied by Dr. Ann Dvorak and according to published methods (31). Tumor specimens obtained at the time of biopsy will be stained for P-Akt and P-MAPK to determine raf-kinase pathway activation. Paraffin-embedded tissue sections will be stained with antibodies against phospho-Akt (P-Akt; Cell Signaling Technology, Beverly, MA) and phospho-MAPK (P-MAPK; Cell Signaling Technology), according to the manufacturer's recommended protocol. Double blind analysis will be carried out for all the markers to ensure objectivity.

7.4. IFP measurements. IFP measurements will be done as previously described (Boucher et al., 1991). To measure IFP a 23 gauge needle with a 3 mm side hole at 5 mm from the tip will be used. Nylon filaments (6-0 ethilon) will be placed in the needle. To take the pressure measurements, the needle will be connected to a pressure transducer by polyethylene tubing filled with sterile heparinized (70 units /ml) saline. The needle and polyethylene tubing will be gas sterilized before use. Before the pressure measurements in each patient, the calibration of the pressure transducer setup will be verified by applying pressures of 5, 10, 20 and 40 mm Hg. Possible leaks in the system will be tested by maintaining the pressure of 40 mm Hg for at least 1 min. With the patient in supine position, the needle will be inserted in the center of the tumor and a pressure recording will be obtained. Stable pressure measurements with a good fluid communication between the tumor interstitial space and needle will be considered valid. The IFP will be measured in three different locations in the tumor and if feasible in the normal tissue surrounding the tumor. For the pressure measurement in normal tissue we will use a needle that has not been introduced in the tumor. Each pressure measurement will require between 5 and 7 min, for a total of approximately 20 min.

The measurements will be performed by either the Attending surgeon (or his/her qualified designee) in the operating room or in a procedure room in the clinic as deemed appropriate. Dr. Yves Boucher (MGH) will teach the technique and quality control assessment to these individuals, although no formal training program/certificate exists.

7.5. Circulating cells and VEGF measurements. Peripheral blood will be collected for the measurements of circulating cells by four-color flow cytometry as described previously (Willett et. al., 2004). Cell suspensions will be evaluated by FACSCalibur (Becton Dickinson, San Jose, CA). CD31 (EC and monocytes), CD45 (pan-hematopoietic marker), CD133 (AC133, progenitor/stem cell marker), and CD34 (progenitor/stem cells, EC) antibodies will be used, and the gate will be set on the lymphocyte/mononuclear populations, to avoid RBCs, cell debris and neutrophil contamination. Data for circulating cells will be obtained as an average of 3 separate measurements, after scanning 50000 events. Fluorescently labeled isotype matched IgG_1 antibodies will be used as control. Soluble VEGF levels in plasma samples will be evaluated from samples obtained throughout the course of the protocol using an ELISA kit (R&D System, Minneapolis).

7.6. ELISA assays will be used to measure the plasma levels of growth factors (VEGF, PDGFA, PDGFB). VEGF levels will also be measured in urine samples by ELISA.

8.0 Surgery

The exact type of surgery for patients in group 1 will be at the discretion of the attending surgeon and appropriate for the individual patient.

9.0 DEFINITIONS OF DISEASE RESPONSE

The primary objectives of this phase II study are to establish the effect of the recommended phase II dose of BAY 43-9006 at 400 mg po two times a day in sarcoma patients on interstitial fluid pressure, blood flow, and parameters on endothelial cell biology.

Any suggestion of antitumor activity is, however, important for the design of further studies and must be described. Response information will be obtained if patients have disease which can be readily measured and reassessed. These assessments will be made every two cycles or more frequently if indicated. Furthermore, a response must be noted with two examinations made four weeks apart in order to be documented as a true response to therapy.

Evaluable for toxicity All patients will be evaluable for toxicity if they receive any study drug.

Evaluable for response All patients who have received a minimum of two cycles of treatment will be considered evaluable for response. In addition, those patients who develop early progressive disease will also be considered evaluable for response. Patients on therapy for at least two cycles of treatment will have their response classified according to the definitions described below.

9.1 Definitions

Response and progression will be evaluated in this study using the new international criteria proposed by the Response Evaluation Criteria in Solid Tumors (RECIST) Committee [*JNCI* 92(3):205-216, 2000]. Changes in only the largest diameter (unidimensional measurement) of the tumor lesions are used in the RECIST criteria. Note: Lesions are either measurable or non-measurable using the criteria provided below. The term “evaluable” in reference to measurability will not be used because it does not provide additional meaning or accuracy.

9.1.1 Measurable Disease

Measurable lesions are defined as those that can be accurately measured in at least one dimension (longest diameter to be recorded) as ≥20 mm with conventional techniques (CT, MRI, X-ray) or as ≥10 mm with spiral CT scan. All tumor measurements must be recorded in millimeters (or decimal fractions of centimeters).

9.1.2 Non-measurable Disease

All other lesions (or sites of disease), including small lesions (longest diameter <20 mm with conventional techniques or <10 mm using spiral CT scan), are considered non-measurable disease. Bone lesions, leptomeningeal disease, ascites, pleural/pericardial effusions, lymphangitis cutis/pulmonis, inflammatory breast disease, abdominal masses (not followed by CT or MRI), and cystic lesions are all non-measurable.

9.1.3 Target Lesions

All measurable lesions up to a maximum of five lesions per organ and 10 lesions in total, representative of all involved organs, should be identified as **target lesions** and recorded and measured at baseline. Target lesions should be selected on the basis of their size (lesions with the longest diameter) and their suitability for accurate repeated measurements (either by imaging techniques or clinically). A sum of the longest diameter (LD) for all target lesions will be calculated and reported as the baseline sum LD. The baseline sum LD will be used as reference by which to characterize the objective tumor response.

9.1.4 Non-target Lesions

All other lesions (or sites of disease) should be identified as **non-target lesions** and should also be recorded at baseline. Non-target lesions include measurable lesions that exceed the maximum numbers per organ or total of all involved organs as well as non-measurable lesions. Measurements of these lesions are not required but the presence or absence of each should be noted throughout follow-up.

9.2 Guidelines for Evaluation of Measurable Disease

All measurements should be taken and recorded in metric notation using a ruler or calipers. All baseline evaluations should be performed as closely as possible to the beginning of treatment and never more than 4 weeks before the beginning of the treatment.

The same method of assessment and the same technique should be used to characterize each identified and reported lesion at baseline and during follow-up. Imaging-based evaluation is preferred to evaluation by clinical examination when both methods have been used to assess the antitumor effect of a treatment.

Clinical lesions. Clinical lesions will only be considered measurable when they are superficial (e.g., skin nodules and palpable lymph nodes). In the case of skin lesions, documentation by color photography, including a ruler to estimate the size of the lesion, is recommended.

Chest X-ray. Lesions on chest X-ray are acceptable as measurable lesions when they are clearly defined and surrounded by aerated lung. However, CT is preferable.

Conventional CT and MRI. These techniques should be performed with cuts of 10 mm or less in slice thickness contiguously. Spiral CT should be performed using a 5 mm contiguous reconstruction algorithm. This applies to tumors of the chest, abdomen, and pelvis. Head and neck tumors and those of extremities usually require specific protocols.

Ultrasound (US). When the primary endpoint of the study is objective response evaluation, US should not be used to measure tumor lesions. It is, however, a possible alternative to clinical measurements of superficial palpable lymph nodes, subcutaneous lesions, and thyroid nodules. US might also be useful to confirm the complete disappearance of superficial lesions usually assessed by clinical examination.

Cytology, Histology. These techniques can be used to differentiate between PR and CR in rare cases (e.g., residual lesions in tumor types such as germ cell tumors, where known residual benign tumors can remain).

The cytological confirmation of the neoplastic origin of any effusion that appears or worsens during treatment when the measurable tumor has met criteria for response or stable disease is mandatory to differentiate between response or stable disease (an effusion may be a side effect of the treatment) and progressive disease.

9.3 Response Criteria

9.3.1 Evaluation of Target Lesions

Complete Response (CR):Disappearance of all target lesions

Partial Response (PR): At least a 30% decrease in the sum of the longest diameter (LD) of target lesions, taking as reference the baseline sum LD

Progressive Disease (PD): At least a 20% increase in the sum of the LD of target lesions, taking as reference the smallest sum LD recorded since the treatment started or the appearance of one or more new lesions

Stable Disease (SD): Neither sufficient shrinkage to qualify for PR nor sufficient increase to qualify for PD, taking as reference the smallest sum LD since the treatment started

9.3.2 Evaluation of Non-target Lesions

Complete Response (CR):	Disappearance of all non-target lesions and normalization of tumor marker level
Incomplete Response/ Stable Disease (SD):	Persistence of one or more non-target lesion(s) or/and maintenance of tumor marker level above the normal limits
Progressive Disease (PD):	Appearance of one or more new lesions and/or unequivocal progression of existing non-target lesions

Note: If tumor markers are initially above the upper normal limit, they must normalize for a patient to be considered in complete clinical response.

9.3.3 Evaluation of Best Overall Response

The best overall response is the best response recorded from the start of the treatment until disease progression/recurrence (taking as reference for progressive disease the smallest measurements recorded since the treatment started). The patient's best response assignment will depend on the achievement of both measurement and confirmation criteria (see section 9.3.1).

9.3.4 Confirmation

The main goal of confirmation of objective response is to avoid overestimating the response rate observed. In cases where confirmation of response is not feasible, it should be made clear when reporting the outcome of such studies that the responses are not confirmed.

To be assigned a status of PR or CR, changes in tumor measurements must be confirmed by repeat assessments that should be performed no less than 4 weeks after the criteria for response are first met. Longer intervals as determined by the study protocol may also be appropriate.

In the case of Stable Disease, follow-up measurements must have met the SD criteria at least once after study entry at a minimum interval (in general, not less than 6-8 weeks) that is defined in the study protocol.

9.3.5 Duration of overall response

The duration of overall response is measured from the time measurement criteria are met for CR or PR (whichever status is recorded first) until the first date that recurrence or PD is objectively documented, taking as reference for PD the smallest measurements recorded since the treatment started.

10.0 Schedule of Assessments

ASSESSMENT	Prestudy (within 14 days of registration (except scans = within 4 weeks)	Each cycle	Weekly[1]	End of Study Visit (within 1 month of last dose[2]	Post-study Followup (one month following last dose)
**Physical examination including a neuro exam, weight and performance status**	X	X	X	X	
Medical history	X	X	X	X	
Sign informed consent	X				
Vital signs	X	X[1]	X		
Toxicity assessment	Continuous				X
**LABORATORY**					
CBC, differential, platelets	X	X	X	X	
Serum chemistry[3]	X	X	X	X	
Pregnancy test[4]	X	X			
EKG	X	X		X	
Urinalysis	X	X			
Pharmacokinetic sampling	Cycle 1: refer to Section 12.0 for schedule				
Functional CT or dce-MRI	Refer to Section 7.1 for schedule				
**STAGING**					
Imaging studies for Tumor Measurements, as appropriate[5] (e.g., CT, MRI, bone scan)	X			X	

1. Weekly during cycle 1, then every other week (cycle 2 through cycle 4), then monthly (cycles 5 and beyond) until off study. Cycle 2 Week 1 there will be an MD visit to review the Day 28 assessment results.
2. Obtained within 1-month post last dose of sorafenib.
3. To include LDH, glucose, electrolytes, albumin, BUN, creatinine, ALT, AST, total bilirubin, Ca^{++}, Mg^{++} and alkaline phosphatase.
4. For women of child-bearing potential (<50 years of age or LMP < 12 months ago), a negative pregnancy test should be documented 72 hours prior to start of treatment with study drug and should be repeated every cycle while patients are on study.
5. Within 4 weeks prior to the start of therapy, repeat as appropriate, every 2 cycles if used to assess tumor measurement.

11.0 Statistical Considerations

Statistical Considerations

Power for the analyses of antivascular markers (the study's primary endpoint) is based on 6 patients with evaluable samples/scans pre- and post- treatment. To achieve that and to evaluate clinical benefit, we plan an overall accrual of 24 patients, of whom 20 are assumed to be eligible.

Primary endpoints: antivascular markers

The goal of this neoadjuvant study is to describe the effect of sorafenib on potentially beneficial antivascular markers in patients with extremity sarcoma. The following table shows key markers, assumptions about the marker at baseline, criteria for rejecting the null hypothesis of no association, statistical test, and power that will exist for a paired comparison using a Wilcoxon signed rank test with one-sided type I error of 5%. We assume that six patients will provide evaluable samples/scans pre- and post-treatment. For Wilcoxon tests, sample size estimates were made using assumptions for the appropriate t-test, then adjusted upward by 16% to account for potential deviations from the normal distribution.

Marker	Assumptions	Target	Power
FDG Uptake (SUVmax)	54% reduction with 38% SD in GIST patients	50% reduction	80%
IFP	15.0 +/- 2.0 pre<br>4.0 +/- 2.2 post in colorectal patients	Change from 15 to 11, SD=3	80%
CEC/WBC	.042 +/- .031 in GIST patients	Change of 0.04, SD 0.03	80%
Pericyte coverage of endothelial cells (α-SMA)	9.9% +/- 3.8% pre<br>17.8% +/- 1.5% post in colorectal patients	Change from 10 to 15%, sd 4%	90%

Overall assessment of clinical benefit

An overall declaration of benefit will be made if the patient has at least one of the following outcomes:

- 50% reduction in interstitial fluid pressure from pre-treatment biopsy to post-treatment biopsy or surgery
- any reduction in tumor dimensions on CT scan as measured by RECIST criteria

We believe these criteria are reasonable in this setting. Changes in tumor appearance on CT scan of this magnitude at approximately 1 month have been shown to be associated with good

clinical outcome in GIST patients. It is hypothesized that lower IFP will reduce the risk of tumor seeding during surgery.

If sorafenib has no antivascular effect, we would expect 5% of patients or fewer to demonstrate benefit from one week of neoadjuvant therapy. Alternatively, if sorafenib acts as hypothesized, the clinical benefit rate could be as high as 25%. We will enroll 20 eligible patients. If 3 or more of 20 eligible patients meet the criteria for clinical benefit, we will consider sorafenib to be efficacious. Using this decision rule, there is 7.5% probability that an ineffective agent will be declared effective (Type I error) and 9.1% probability that an effective agent will be found to have no activity (power of about 91%).

Adverse event evaluation

All patients who receive a single dose of sorafenib, regardless of eligibility, will be included in the assessment of adverse events. Assuming all 24 patients are treated, a 90% confidence interval on the rate of severe toxicities will be no wider than 36% (+/- 18%). The probability of observing a rare toxicity (true population rate of 5%) will be about 71%.

12.0 Pharmacokinetic Studies

The pharmacokinetics of BAY 43-9006 will be determined in all patients entered into the study during the first cycle of therapy. Sampling will be performed on day 14 for patients in treatment group I and on days 28 and 56 for patients in treatment group II. The concentration of the drug will be measured in plasma specimens by high-performance liquid chromatography with electrospray ionization mass spectrometric detection. The objective of the pharmacokinetic study is to assess the existence of correlations between pharmacokinetic variablesand results of the other correlative studies that are being performed. The sampling schedule and summary of procedures that will be used to establish times, collect samples, and process specimens for storage prior to analysis, to insure the acquisition of accurate pharmacokinetic data, are described below.

The sampling schedule has been devised to accommodate treatment on an outpatient basis. Ingestion of the drug must be started not later than 10:00 a.m. on Monday, Tuesday, or Wednesday to facilitate the collection of the pharmacokinetic samples at the scheduled times during the regular operating hours of the outpatient clinics. Before starting the drug on the days that pharmacokinetic samples will be collected, place a large gauge peripheral catheter (*e.g.* 19 or 20 gauge Angiocath straight set with T-connector, or similar IV access device) within a vein of the arm that is not receiving the infusion line. This catheter will be used for pharmacokinetic blood sample collection and should be maintained patent between blood draws with a heparin lock (10 U/mL in normal saline) or a slow drip of Normal Saline for Injection, USP (10 mL/hr). Pharmacokinetic blood specimens may be obtained by venipuncture, without the use of a catheter, on days when only 1-2 samples are scheduled to be drawn.

Verify the operation of a battery-powered digital timer/stopwatch and program it to operate as a 24 hr clock. The same timer must be allowed to run without interruption until the last blood specimen has been obtained from the patient. Timer readings will be noted at the precise time

that the infusion is started and ended, as well as at the beginning and ending times of the blood sample collection intervals. Readings of the digital timer must be directly recorded on a copy of the appropriate 'DF/HCC Pharmacokinetic Dosing and Blood Collection Time Form', prepared specifically. An electronic copy of this form will be posted on the following PHS shared directory: W:\'PHASE1RD'.

Blood specimens (7 mL) will be drawn at the following times relative to the ingestion of BAY 43-9006 :

t_1 = 0 (shortly before dosing)
t_2 = 30 min
t_3 = 1 h
t_4 = 2 h
t_5 = 4 h
t_6 = 6 h
t_7 = 8 h
t_8 = 24 h

The following procedures will be used to collect, process and store the pharmacokinetic blood samples:

Begin to clear the catheter approximately 1 min before the specified sample time by withdrawing the lock solution and approximately 0.5 mL of blood into a syringe. Remove and properly dispose the syringe used to clear the catheter. Draw 7 ml of blood into a green stoppered Vacutainer plasma collection tube with freeze dried sodium heparin (Becton-Dickinson). Promptly mix the plasma collection tube by gently inverting 6-times, then place it on wet ice, and centrifuge (1,100-1,300 x g, 10 min, 4°C) within 5-10 min after collection. Separate the plasma from the blood cells using a pipette and transfer it into a 4.5 mL self-standing polypropylene cryogenic tube with external threads. Affix a computed printed label (obtain file on the W:PHASE1RD directory) to the cryotube, oriented lengthwise toward the upper part of the tube. Completely cover the label with protective cryogenic freezer tape. Place the tube on crushed dry-ice until stored in a freezer maintained at ≤ -70°C. Deliver the samples, packaged in crushed dry-ice, to the Clinical Pharmacology Laboratory (MGH, GRJ 1025, phone 726-5854), together with the time sheet, for HPLC analysis.

The total volume of blood that is scheduled to be collected from each patient for pharmacokinetic studies during the first cycle of therapy is 48-64 mL. The sample collection times and/or the number of samples collected may be modified, at the discretion of the Study Chair, as pharmacokinetic data becomes available during the cycle of the trial to insure accurate definition of the plasma concentration-time profiles. However, the cumulative volume of blood collected for pharmacokinetic studies may not exceed 200 mL, which is < 5% of the total blood volume for a 60 kg patient, during a 28 day treatment cycle.

Time points will be determined as the difference between the midpoint of the blood collection interval and starting time of dose administration. Concentration-time profiles of BAY 43-9006 and its metabolites will be analyzed by noncompartmental methods and/or nonlinear least squares regression using WinNonlin (Scientific Consulting, Inc.). Pharmacokinetic parameters

and variables will be calculated according to standard equations. Mean values of pharmacokinetic parameters will be statistically compared using the two-tailed t-test of the log-transformed data.

13.0 Monitoring and Quality Assurance

13.1 Informed Consent

The principles of informed consent are described by Federal Regulatory Guidelines (Federal Register Vol 46, No. 17, January 27, 1981 part 50). They must be followed to comply with FDA and OPRR regulations for the conduct and monitoring of clinical investigations.

13.2 Institutional Reviews

This study must be approved by an appropriate institutional review committee as defined by Federal Guidelines (Federal Register Vol 46, No. 17, January 27, 1981 part 56).

13.3 Publication of Data

The investigators maintain the full right to publish and present any data obtained during the course of this clinical trial.

13.4 Inclusion of Women and Minorities

This study is not designed to measure the differences in intervention effect but the inclusion and reporting of information on women and minorities will contribute to an increase in the scientific base of knowledge which will aid in planning future clinical trials. The participation of the consortium members (MGH, DFCI, BWH) will serve as the recruitment mechanism for enrollment on this clinical trial. The racial composition of our service area is 88% white, 5% black, 5% Hispanic and 2% Asian and others. The breakdown by gender is approximately 50% male and 50% female.

14.0 Cooperative Research and Development Agreement (CRADA)/Clinical Trials Agreement (CTA)

The agent(s), supplied by CTEP, DCTD, NCI, used in this protocol is/are provided to the NCI under a Collaborative Agreement (CRADA, CTA) between the Pharmaceutical Company(ies) [hereinafter referred to as ACollaborator(s)@] and the NCI Division of Cancer Treatment and Diagnosis. Therefore, the following obligations/guidelines, in addition to the provisions in the AIntellectual Property Option to Collaborator@ contained within the terms of award, apply to the use of Agent(s) in this study:

1. Agent(s) may not be used for any purpose outside the scope of this protocol, nor can Agent(s) be transferred or licensed to any party not participating in the

clinical study. Collaborator(s) data for Agent(s) are confidential and proprietary to Collaborator(s) and shall be maintained as such by the investigators. The protocol documents for studies utilizing investigational agents contain confidential information and should not be shared or distributed without the permission of the NCI. If a copy of this protocol is requested by a patient participating on the study or patient's family member, the individual should sign a confidentiality agreement. A suitable model agreement can be downloaded from http://ctep.cancer.gov.

2. For a clinical protocol where there is an investigational Agent used in combination with (an)other investigational Agent(s), each the subject of different collaborative agreements, the access to and use of data by each Collaborator shall be as follows (data pertaining to such combination use shall hereinafter be referred to as "Multi-Party Data".):

    a. NCI must provide all Collaborators with prior written notice regarding the existence and nature of any agreements governing their collaboration with NIH, the design of the proposed combination protocol, and the existence of any obligations that would tend to restrict NCI's participation in the proposed combination protocol.

    b. Each Collaborator shall agree to permit use of the Multi-Party Data from the clinical trial by any other Collaborator solely to the extent necessary to allow said other Collaborator to develop, obtain regulatory approval, or commercialize its own investigational agent.

    c. Any Collaborator having the right to use the Multi-Party Data from these trials must agree in writing prior to the commencement of the trials that it will use the Multi-Party Data solely for development, regulatory approval, and commercialization of its own investigational agent.

3. Clinical Trial Data and Results and Raw Data developed under a collaborative agreement will be made available exclusively to Collaborator(s), the NCI, and the FDA, as appropriate. All data made available will comply with HIPAA regulations.

4. When a Collaborator wishes to initiate a data request, the request should first be sent to the NCI, who will then notify the appropriate investigators (Group Chair for Cooperative Group studies, or PI for other studies) of Collaborator's wish to contact them.

5. Any data provided to Collaborator(s) for phase 3 studies must be in accordance with the guidelines and policies of the responsible Data Monitoring Committee (DMC), if there is a DMC for this clinical trial.

6. Any manuscripts reporting the results of this clinical trial must be provided to CTEP for immediate delivery to Collaborator(s) for advisory review and comment prior to submission for publication. Collaborator(s) will have 30 days from the date of receipt for review. Collaborator shall have the right to request that publication be delayed for up to an additional 30 days in order to ensure that Collaborator's confidential and proprietary data, in addition to Collaborator(s)'s intellectual property rights, are protected. Copies of abstracts must be provided to CTEP for forwarding to Collaborator(s) for courtesy review as soon as possible and preferably at least three (3) days prior to submission, but in any case, prior to presentation at the meeting or publication in the proceedings. Press releases and other media presentations must also be forwarded to CTEP prior to release. Copies of any manuscript, abstract, and/or press release/ media presentation should be sent to:

   Regulatory Affairs Branch, CTEP, DCTD, NCI
   6130 Executive Boulevard, Suite 7111
   Rockville, MD 20852
   FAX 301-402-1584
   E-mail: anshers@ctep.nci.nih.gov

   The Regulatory Affairs Branch will then distribute them to Collaborator(s). No publication, manuscript or other form of public disclosure shall contain any of Collaborator's confidential/proprietary information.

15.0 References

[1] Gullino,P, Clark S,& Grantham F. *Cancer Research, 24:780-797, 1964.*

[2] Boucher, Y., Baxter, L., and Jain, R. K. *Cancer Res., 50: 4478–4484, 1990*

[3] Leu A, Berk DA, Lymboussaki A, Alitalo K, and. Jain RK**.** *Cancer Research 60, 4324–4327, 2000.*

[4] Baxter, L. T., and Jain, RK. *Microvasc. Res., 37: 77–104, 1989.*

[5] Milosevic, M, Fyles, A W, and Hill, RP. Int. J. Radiat. Oncol. Biol. Phys., 43: 1111–1123, 1999.

[6] Milosevic M, Fyles A, Hedley D, Pintilie M, Levin W, Manchul L, and Hill R. *Cancer Research 61: 6400-6405, 2001.*

[7] Ferrara N, Gerber H-P, LeCouter J, and Lin R. *Nat Med 9:669-6676, 2003.*

[8] Pietras, K., Ostman, A., Sjoquist, M., Buchdunger, E., Reed, R. K., Heldin, C-H., and Rubin, K. Cancer Res., *61:2929–2934, 2001.*

[9] Pietras K, Rubin K, Sjöblom T, Buchdunger E, Sjöquist M, Heldin K-H, and Östman A. *Cancer Research 62:5476-5484, 2002.*

[10] Gordon MS, Margolin K, Talpaz M, et al:. *J Clin Oncol 19:843-850, 2001.*

[11] Hurwitz H, Fehrenbacher L, Cartwright T, Hainsworth J, Heim W, Berlin J, Griffing S, Novotny W, Holmgren E, and Kabbinavar F. *Proc. ASCO 39: 3646 (abstract), 2003.*

[12] Less JR, Posner MC, Boucher Y, et al: "Interstitial Hypertension in Human Breast and Colorectal Tumors," Cancer Res., 52: 6371-6374, 1992.

[13] Jain R.K. Normalizing tumur vasculature with anti-angiogenic therapy: A new paradigm for combination therapy. Nat Med., 7:987-989, 2001

[14] Willett CG, Boucher Y, di Tomaso E, Duda DG, Munn LL, Tong RT, Chung DC, Sahani DV, Kalva SP, Kozin SV, Mino M, Cohen KS, Scadden DT, Hartford AC, Fischman AJ, Clark JW, Ryan DP, Zhu AX, Blaszkowsky LS, Chen HX, Shellito PC, Lauwers GY, Jain RK. Direct evidence that the VEGF-specific antibody bevacizumab has antivascular effects in human rectal cancer. Nat. Med. 10: 145-147, 2004.

[15] Tong RT, Boucher Y, Kozin SV, Winkler F, Hicklin DJ, Jain RK. Vascular normalization by vascular endothelial growth factor receptor 2 blockade induces a pressure gradient across the vasculature and improves drug penetration in tumors. Cancer Res. 64:3731-3736, 2004.

[16] Weidner N. Angiogenesis as a predictor of clinical outcome in cancer patients. Hum. Pathol., 31: 403-405, 2000.

[17] Yudoh K, Kanamori M, Ohmori K, Yasuda T, Aoki M, Kimura T. Concentration of vascular endothelial growth factor in the tumour tissue as a prognostic factor of soft tissue sarcomas. Brit. J. Cancer 84:1610-5, 2001.

[18] Monestiroli S, Mancuso P, Burlini A, Pruneri G, Dell'Agnola C, Gobbi A, Martinelli G, Bertolini F. Kinetics and viability of circulating endothelial cells as surrogate angiogenesis marker in an animal model of human lymphoma. Cancer Res., 61:4341-4344, 2001

[19] Mancuso P., Burlini A., Pruneri G., Goldhirsch A., Martinelli G., Bertolini F. Resting and activated endothelial cells are increased in the peripheral blood of cancer patients. Blood, 97: 3658-3661, 2001.

[20] Chang Y.S., di Tomaso E., McDonald D.M., Jones R., Jain R.K., Munn L.L. Mosaic blood vessels in tumors: frequency of cancer cells in contact with flowing blood. Proc. Natl. Acad. Sci. USA, 97:14608-14613, 2000.

[21] Lyden D., Hattori K., Dias S., Costa C., Blaikie P., Butros L., Chadburn A., Heissig B., Marks W., Witte L., Wu Y., Hicklin D., Zhu Z., Hackett N.R., Crystal R.G., Moore M.A., Hajjar K.A., Manova K., Benezra R., Rafii S. Impaired recruitment of bone-marrow-derived endothelial and hematopoietic precursor cells blocks tumor angiogenesis and growth. Nat. Med., 7:1194-201, 2001.

[22] Roh HD, Boucher Y, Kalnicki S, et al: Interstitial hypertension in carcinoma of the uterine cervix in patients: Possible correlation with tumor oxygenation and radiation response. Cancer Res., 51: 6695-6698, 1991.

[23]Boucher Y, Kirkwood JM, Opacic D, Jain RK. Interstitial hypertension in superficial metastatic melanomas in humans. Cancer Res. 51: 6691-6694, 1991.

[24] Less JR, Posner MC, Boucher Y, et al: "Interstitial Hypertension in Human Breast and Colorectal Tumors," Cancer Res., 52: 6371-6374, 1992.

[25] Boucher Y, Salehi H, Witwer B, et al: Interstitial fluid pressure in intracranial tumors in patients and in rodents. Brit. J. Cancer 75: 829-836, 1997.

[26] Milosevic MF, Fyles AW, Wong R, et al: Interstitial Fluid Pressure in Cervical Carcinoma: Within Tumor Heterogeneity, and Relation to Oxygen Tension, Cancer 82: 2418-2426, 1998.

[27] Milosevic M, Fyles A, Hedley D, et al: Interstitial Fluid Pressure Predicts Survival in Patients with Cervix Cancer Independent of Clinical Prognostic Factors and Tumor Oxygen Measurements. Cancer Res., 61:6400-6405, 2001.

[28] Lee CG, Heijn M, di Tomaso E, et al: Anti-Vascular Endothelial Growth Factor Treatment Augments Tumor Radiation Response under Normoxic or Hypoxic Conditions. Cancer Res., 60:5565-5570, 2000.

[30]Avali A, Hood JD, Frausto R, Stupack DG, and Cheresh DA. Role of *raf* in vascular protection from distinct apoptotic stimuli. *Science 301: 94-96, 2003.*

[31] Feng D, Nagy JA, Brekken RA, Pettersson A, Manseau EJ, Pyne K, Mulligan R, Thorpe PE, Dvorak HF, and Dvorak AM **.** Ultrastructural Localization of the Vascular Permeability Factor/Vascular Endothelial Growth Factor (VPF/VEGF) Receptor-2 (FLK-1, KDR) in Normal Mouse Kidney and in the Hyperpermeable Vessels Induced by VPF/VEGF-expressing Tumors and Adenoviral Vectors. The Journal of Histochemistry & Cytochemistry Volume 48(4): 545–555, 2000

Comprehensive Adverse Events and Potential Risks List (CAEPR) for Sorafenib (BAY 43-9006, NSC 724772)

The Comprehensive Adverse Event and Potential Risks list (CAEPR) provides a single, complete list of reported and/or potential adverse events (AE) associated with an agent using a uniform presentation of events by body system. In addition to the comprehensive list, a subset, the Agent Specific Adverse Event List (ASAEL), appears in a separate column and is identified with ***bold*** and ***italicized*** text. This subset of AEs (ASAEL) contains events that are considered 'expected' for expedited reporting purposes only. Refer to the 'CTEP, NCI Guidelines: Adverse Event Reporting Requirements' http://ctep.cancer.gov/reporting/adeers.html for further clarification. *Frequency is provided based on 1376 patients*. Below is the CAEPR for sorafenib.

Version 2.0, February 22, 2006[1]

Adverse Events with Possible Relationship to Sorafenib (BAY 43-9006) (CTCAE v3.0 Term) [n=1376 patients]			'Agent Specific Adverse Event List' (ASAEL)
**Likely (>20%)**	**Less Likely (<=20%)**	**Rare but Serious (<3%)**	
ALLERGY/IMMUNOLOGY			
	Allergic reaction/hypersensitivity (including drug fever)		
BLOOD/BONE MARROW			
	Hemoglobin		
	Leukocytes (total WBC)		
	Lymphopenia		
	Neutrophils/granulocytes (ANC/AGC)		
	Platelets		
CARDIAC GENERAL			
	Hypertension		***Hypertension***
CONSTITUTIONAL SYMPTOMS			
Fatigue (asthenia, lethargy, malaise)			***Fatigue (asthenia, lethargy, malaise)***
	Fever (in the absence of neutropenia, where neutropenia is defined as ANC <1.0 x 10e9/L)		
	Rigors/chills		
	Weight loss		
DERMATOLOGY/SKIN			
	Dry skin		
	Flushing		
	Hair loss/alopecia (scalp or body)		
	Hypopigmentation		
	Nail changes		
	Pruritus/itching		
Rash/desquamation			***Rash/desquamation***
Rash: hand-foot skin reaction			***Rash: hand-foot skin reaction***
	Dermatology/skin – Other: non-life threatening squamous cell carcinoma of the skin: keratoacanthoma type		

GASTROINTESTINAL			
	Anorexia		
	Ascites (non-malignant)		
	Constipation		
	Dehydration		
Diarrhea			***Diarrhea***
	Dysphagia (difficulty swallowing)		
	Flatulence		
	Heartburn/dyspepsia		
	Mucositis/stomatitis (functional/symptomatic): pharynx		
	Nausea		
	Vomiting		
HEMORRHAGE/BLEEDING			
	Hemorrhage GI - Select		
	Hemorrhage GU - Select		
HEPATOBILIARY/PANCREAS			
	Pancreatitis		
INFECTION			
	Febrile neutropenia (fever of unknown origin without clinically or microbiologically documented infection)(ANC <1.0 x 10e9/L, fever >=38.5 degrees C)		
	Infection with unknown ANC-Select		
METABOLIC/LABORATORY			
	Albumin, serum-low (hypoalbuminemia)		
	Alkaline phosphatase		
	ALT, SGPT (serum glutamic pyruvic transaminase)		
	Amylase		
	AST, SGOT (serum glutamic oxaloacetic transaminase)		
	Bilirubin (hyperbilirubinemia)		
	GGT (gamma-glutamyl transpeptidase)		
	Glucose, serum-high (hyperglycemia)		
	Phosphate, serum-low (hypophosphatemia)		
	Lipase		
	Metabolic/Laboratory - Other (blood elastase)		
NEUROLOGY			
	Neuropathy: sensory		
PAIN			
	Pain - abdomen NOS		
	Pain - joint		
	Pain - muscle		
	Pain NOS		

PULMONARY/UPPER RESPIRATORY			
	Hypoxia		
	Pleural effusion (non-malignant)		
	Pneumonitis/pulmonary infiltrates		
	Pneumothorax		
RENAL/GENITOURINARY			
	Renal failure		
SYNDROMES			
	Flu-like syndrome		

[1]This table will be updated as the toxicity profile of the agent is revised. Updates will be distributed to all Principal Investigators at the time of revision. The current version can be obtained by contacting ADEERSMD@tech-res.com. Your name, the name of the investigator, the protocol and the agent should be included in the e-mail.

Also reported on sorafenib trials but with the relationship to sorafenib still undetermined:
ALLERGY/IMMUNOLOGY - linear IgA disease
CARDIAC ARRHYTHMIA - atrial flutter; supraventricular arrhythmia
CARDIAC GENERAL - cardiac ischemia/infarction; left ventricular systolic dysfunction
COAGULATION - INR; PTT; thrombotic microangiopathy
DERMATOLOGY/SKIN – erythema multiforme
GASTROINTESTINAL - ileus
HEMORRHAGE/BLEEDING - CNS hemorrhage; petechiae; pleural hemorrhage; splenic infarction
LYMPHATICS - limb edema
METABOLIC/LABORATORY - creatinine; hyperuricemia; hyponatremia
MUSCULOSKELETAL/SOFT TISSUE - arthritis
NEUROLOGY - anxiety; CNS ischemia; dizziness; encephalopathy; memory impairment; psychosis; syncope
OCULAR/VISUAL - diplopia; uveitis
PAIN - back pain; bone pain; chest/thorax pain; headache; limb pain
PULMONARY/UPPER RESPIRATORY - ARDS; dyspnea; voice changes
SEXUAL/REPRODUCTIVE FUNCTION – erectile dysfunction
VASCULAR - thrombosis/thrombus/embolism

Note: Sorafenib in combination with other agents could cause an exacerbation of any adverse event currently known to be caused by the other agent, or the combination may result in events never previously associated with either agent.

Dana-Farber Harvard Cancer Center

DF/HCC Protocol # 05-033
(NCI Protocol 6948)

Study Medication Diary

TREATMENT GROUP: ___________

Cycle ________

Name:____

Number:______________________

****
Please bring this diary to each clinic visit
****

Comments Log

Please explain any missed doses or dosing complications for incomplete doses below

Study Day	Comments – Missed and Incomplete doses
**1**	
**2**	
**3**	
**4**	
**5**	
**6**	
**7**	
**8**	
**9**	
**10**	
**11**	
**12**	
**13**	
**14**	

Instructions:

STUDY MEDICATION IS STORED AT ROOM TEMPERATURE

- **You must take your medication twice a day, 2 tablets in the morning and 2 tablets in the evening, with a glass of water. You should avoid eating a high fat meal around the time that you take your study medication.**
- **Tablets should be taken with enough water to assist with easy swallowing of each tablet.**
- For doses taken at home, please indicate if the dose was taken (yes or no) on the appropriate day as listed on the diary log.
  - o If a dose was not taken, please provide an explanation for the missed dose (e.g., “bottle lost”, “dose forgotten”, etc.) on the Dosing Comments page in the space provided for that study day.

    If you miss a dose or vomit a dose, <u>do not make it up</u>. Resume taking sorafenib at the next scheduled dose.
- Record the date and time of each dose taken.
  - o Please indicate AM or PM for the time
- Indicate whether there were problems with a dose taken (e.g., you experience nausea and vomiting within 30 minutes of dosing).
  - o If you experience any problems after dosing, please provide an explanation on the Dosing Comments page in the space provided for that study day.
- Please bring your study medication diary with you to each clinic visit.

Dosing Diary

Study Day	Dose taken? *If no, explain on next page*	Was the dose complete? *If no, explain on next page.*
1	☐ Yes ☐ No	☐ Yes ☐ No
2	☐ Yes ☐ No	☐ Yes ☐ No
3	☐ Yes ☐ No	☐ Yes ☐ No
4	☐ Yes ☐ No	☐ Yes ☐ No
5	☐ Yes ☐ No	☐ Yes ☐ No
6	☐ Yes ☐ No	☐ Yes ☐ No
7	☐ Yes ☐ No	☐ Yes ☐ No
8	☐ Yes ☐ No	☐ Yes ☐ No
9	☐ Yes ☐ No	☐ Yes ☐ No
10	☐ Yes ☐ No	☐ Yes ☐ No
11	☐ Yes ☐ No	☐ Yes ☐ No
12	☐ Yes ☐ No	☐ Yes ☐ No
13	☐ Yes ☐ No	☐ Yes ☐ No
14	☐ Yes ☐ No	☐ Yes ☐ No

Date of Dose<br>MM DD YY	Time of Dose<br>Morning	Evening
☐☐ ☐☐ 2 0 ☐☐	☐☐:☐☐	☐☐:☐☐
☐☐ ☐☐ 2 0 ☐☐	☐☐:☐☐	☐☐:☐☐
☐☐ ☐☐ 2 0 ☐☐	☐☐:☐☐	☐☐:☐☐
☐☐ ☐☐ 2 0 ☐☐	☐☐:☐☐	☐☐:☐☐
☐☐ ☐☐ 2 0 ☐☐	☐☐:☐☐	☐☐:☐☐
☐☐ ☐☐ 2 0 ☐☐	☐☐:☐☐	☐☐:☐☐
☐☐ ☐☐ 2 0 ☐☐	☐☐:☐☐	☐☐:☐☐
☐☐ ☐☐ 2 0 ☐☐	☐☐:☐☐	☐☐:☐☐
☐☐ ☐☐ 2 0 ☐☐	☐☐:☐☐	☐☐:☐☐
☐☐ ☐☐ 2 0 ☐☐	☐☐:☐☐	☐☐:☐☐
☐☐ ☐☐ 2 0 ☐☐	☐☐:☐☐	☐☐:☐☐
☐☐ ☐☐ 2 0 ☐☐	☐☐:☐☐	☐☐:☐☐
☐☐ ☐☐ 2 0 ☐☐	☐☐:☐☐	☐☐:☐☐
☐☐ ☐☐ 2 0 ☐☐	☐☐:☐☐	☐☐:☐☐

Study Day	Doses taken? *If no, explain on next page*	Were the doses complete? *If no, explain on next page.*
15	☐ Yes ☐ No	☐ Yes ☐ No
16	☐ Yes ☐ No	☐ Yes ☐ No
17	☐ Yes ☐ No	☐ Yes ☐ No
18	☐ Yes ☐ No	☐ Yes ☐ No
19	☐ Yes ☐ No	☐ Yes ☐ No
20	☐ Yes ☐ No	☐ Yes ☐ No
21	☐ Yes ☐ No	☐ Yes ☐ No
22	☐ Yes ☐ No	☐ Yes ☐ No
23	☐ Yes ☐ No	☐ Yes ☐ No
24	☐ Yes ☐ No	☐ Yes ☐ No
25	☐ Yes ☐ No	☐ Yes ☐ No
26	☐ Yes ☐ No	☐ Yes ☐ No
27	☐ Yes ☐ No	☐ Yes ☐ No
28	☐ Yes ☐ No	☐ Yes ☐ No

Date of Dose MM DD YY	Time of Dose	
☐☐ ☐☐ 2 0 ☐☐	☐☐:☐☐	☐☐:☐☐
☐☐ ☐☐ 2 0 ☐☐	☐☐:☐☐	☐☐:☐☐
☐☐ ☐☐ 2 0 ☐☐	☐☐:☐☐	☐☐:☐☐
☐☐ ☐☐ 2 0 ☐☐	☐☐:☐☐	☐☐:☐☐
☐☐ ☐☐ 2 0 ☐☐	☐☐:☐☐	☐☐:☐☐
☐☐ ☐☐ 2 0 ☐☐	☐☐:☐☐	☐☐:☐☐
☐☐ ☐☐ 2 0 ☐☐	☐☐:☐☐	☐☐:☐☐
☐☐ ☐☐ 2 0 ☐☐	☐☐:☐☐	☐☐:☐☐
☐☐ ☐☐ 2 0 ☐☐	☐☐:☐☐	☐☐:☐☐
☐☐ ☐☐ 2 0 ☐☐	☐☐:☐☐	☐☐:☐☐
☐☐ ☐☐ 2 0 ☐☐	☐☐:☐☐	☐☐:☐☐
☐☐ ☐☐ 2 0 ☐☐	☐☐:☐☐	☐☐:☐☐
☐☐ ☐☐ 2 0 ☐☐	☐☐:☐☐	☐☐:☐☐
☐☐ ☐☐ 2 0 ☐☐	☐☐:☐☐	☐☐:☐☐
